# Supplementary material for: Development of Phosphoramidite Reagents for the Synthesis of Base-Labile Oligonucleotides Modified with a Linear Aminoalkyl and Amino-PEG Linker at the 3′-End
Source: Molecules. 2022 Dec 3;27(23):8501. doi: 10.3390/molecules27238501 (PMC9736658; doi:10.3390/molecules27238501)
Supplement: Supplementary file 1 [file molecules-27-08501-s001.zip › molecules-2063086-supplementary.pdf]

Supporting Information

Development of phosphoramidite reagents for the synthesis of base-labile oligonucleotides modified with a linear aminoalkyl and amino-PEG linker at the 3′-end

Takashi Osawa,[a] Qin Ren,[a] and Satoshi Obika*,[a,b,c]

[a]*Graduate School of Pharmaceutical Sciences, Osaka University, Yamadaoka 1-6, Suita, Osaka 565-0871, Japan.*

[b]*Institute for Open and Transdisciplinary Research Initiatives, Osaka University, Yamadaoka 1-3, Suita, Osaka 565-0871, Japan.*

[c]*National Institutes of Biomedical Innovation, Health and Nutrition, 7-6-8 Saito-Asagi, Ibaraki, Osaka 567-0085, Japan.*

Contents:

- **Figure S1**. ^{1}H NMR and ^{13}C NMR spectra of compound **2** S2
- **Figure S2**. ^{1}H NMR and ^{13}C NMR spectra of compound **4a** S3
- **Figure S3**. ^{1}H NMR and ^{13}C NMR spectra of compound **4b** S4
- **Figure S4**. ^{1}H NMR and ^{13}C NMR spectra of compound **4c** S5
- **Figure S5**. ^{1}H NMR and ^{13}C NMR spectra of compound **5a** S6
- **Figure S6**. ^{1}H NMR and ^{13}C NMR spectra of compound **5b** S7
- **Figure S7**. ^{1}H NMR and ^{13}C NMR spectra of compound **5c** S8
- **Figure S8**. ^{1}H NMR and ^{31}P NMR spectra of compound **6a** S9
- **Figure S9**. ^{1}H NMR and ^{31}P NMR spectra of compound **6b** S10
- **Figure S10**. ^{1}H NMR and ^{31}P NMR spectra of compound **6c** S11
- **Figure S11**. LC/MS charts of crude C10-mer oligonucleotides S12
- **Figure S12**. LC/MS charts of crude 13-mer oligonucleotides with a 3′-aminopropyl linker S13
- **Figure S13**. LC/MS charts of crude 13-mer oligonucleotides with a 3′-aminohexyl linker S14
- **Figure S14**. LC/MS charts of crude 13-mer oligonucleotides with a 3′-amino-PEG2 linker S15
- **Figure S15**. LC/MS charts of crude IdU-modified oligonucleotides with a 3′-aminopropyl linker S16
- **Figure S16**. LC/MS charts of crude AmNA-modified oligonucleotides with a 3′-aminopropyl linker S17
- **Figure S17**. LC/MS charts of crude TAMRA-T-modified oligonucleotides with a 3′-aminopropyl linker S18
- **Figure S18**. LC/MS charts of crude IdU-modified oligonucleotides with a 3′-aminohexyl and amino-PEG2 linker S19
- **Figure S19**. LC/MS charts of crude AmNA-modified oligonucleotides with a 3′-aminohexyl and amino-PEG2 linker S20
- **Figure S20**. LC/MS charts of crude TAMRA-T-modified oligonucleotides with a 3′-aminohexyl and amino-PEG2 linker S21

^{1}H NMR ($CDCl_3$)

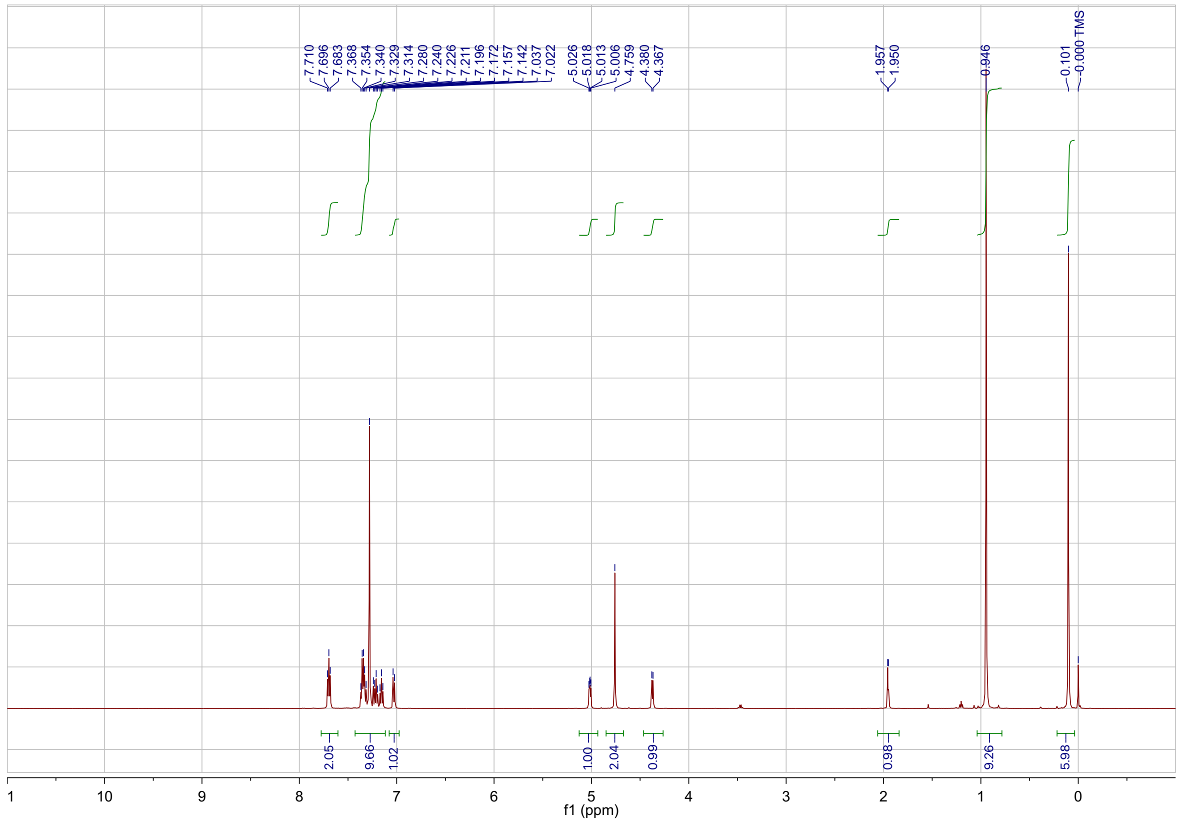

^{13}C NMR ($CDCl_3$)

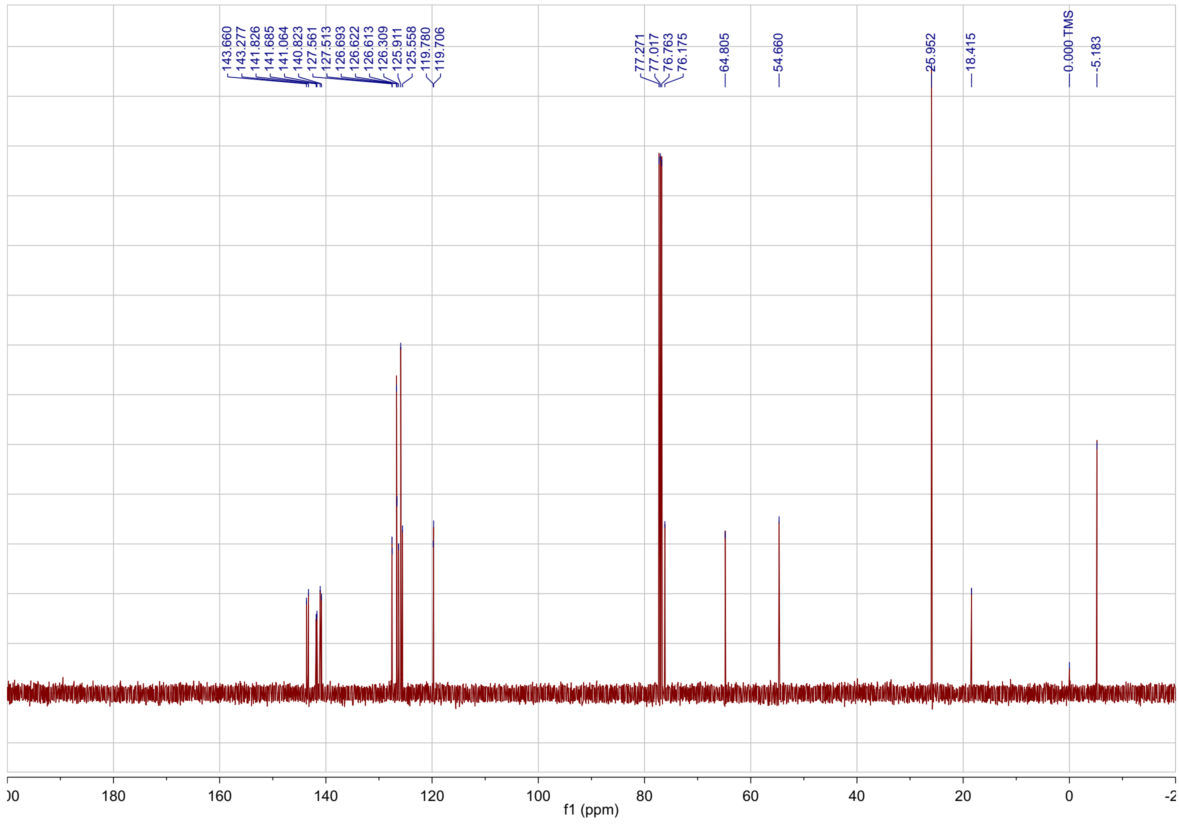

Figure S1. ^{1}H NMR and ^{13}C NMR spectra of compound **2**

^{1}H NMR ($CDCl_3$)

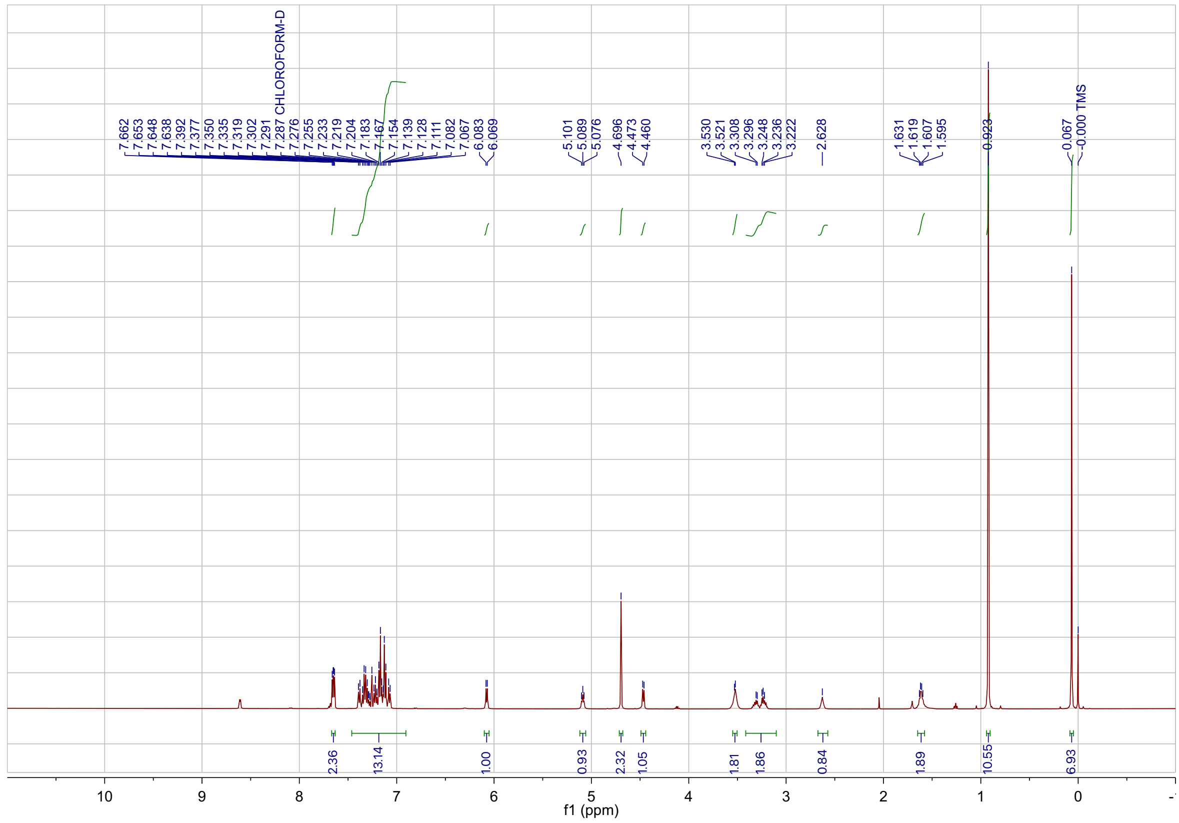

^{13}C NMR ($CDCl_3$)

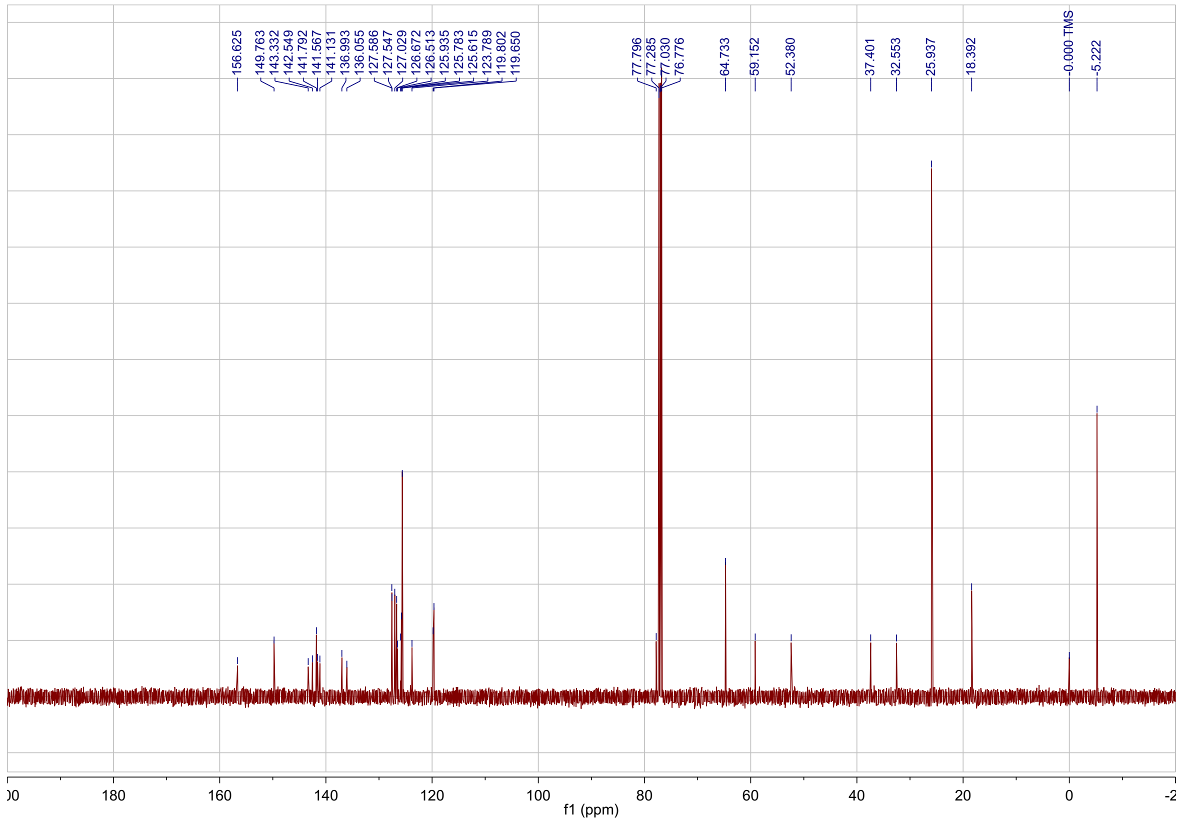

Figure S2. ^{1}H NMR and ^{13}C NMR spectra of compound **4a**

^{1}H NMR ($CDCl_3$)

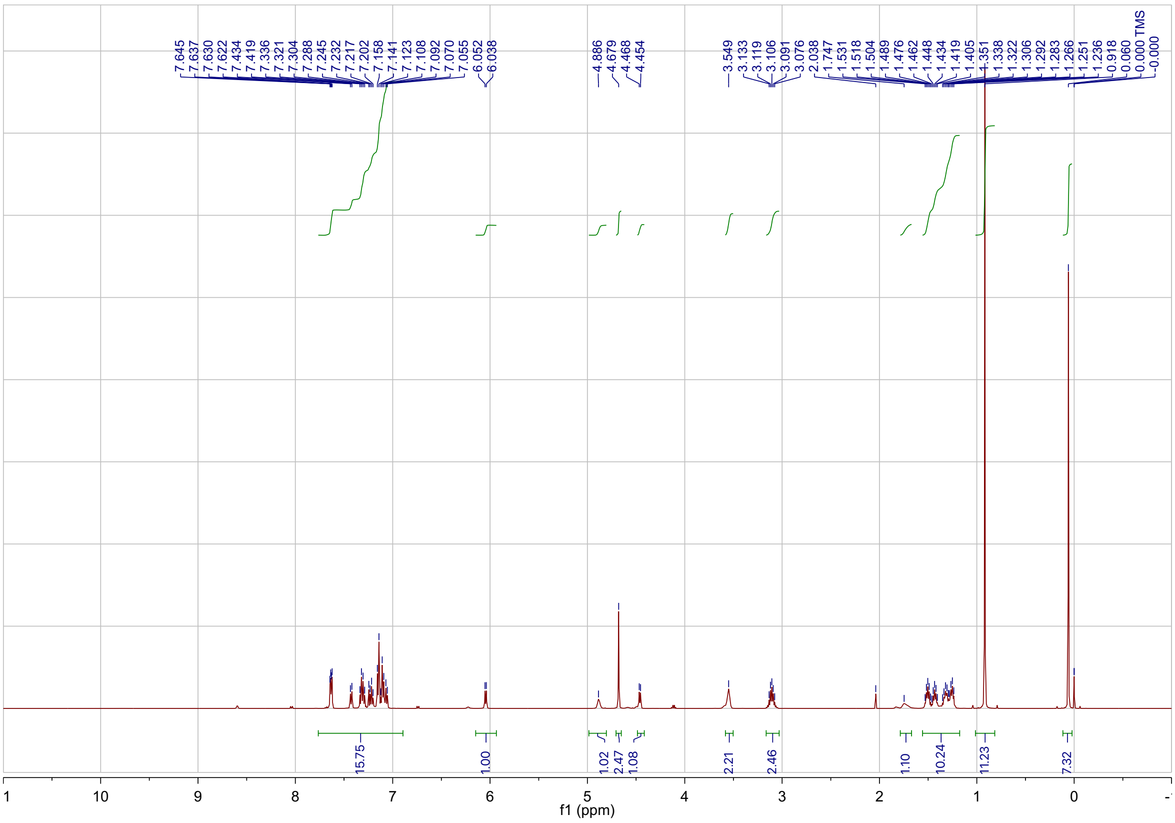

^{13}C NMR ($CDCl_3$)

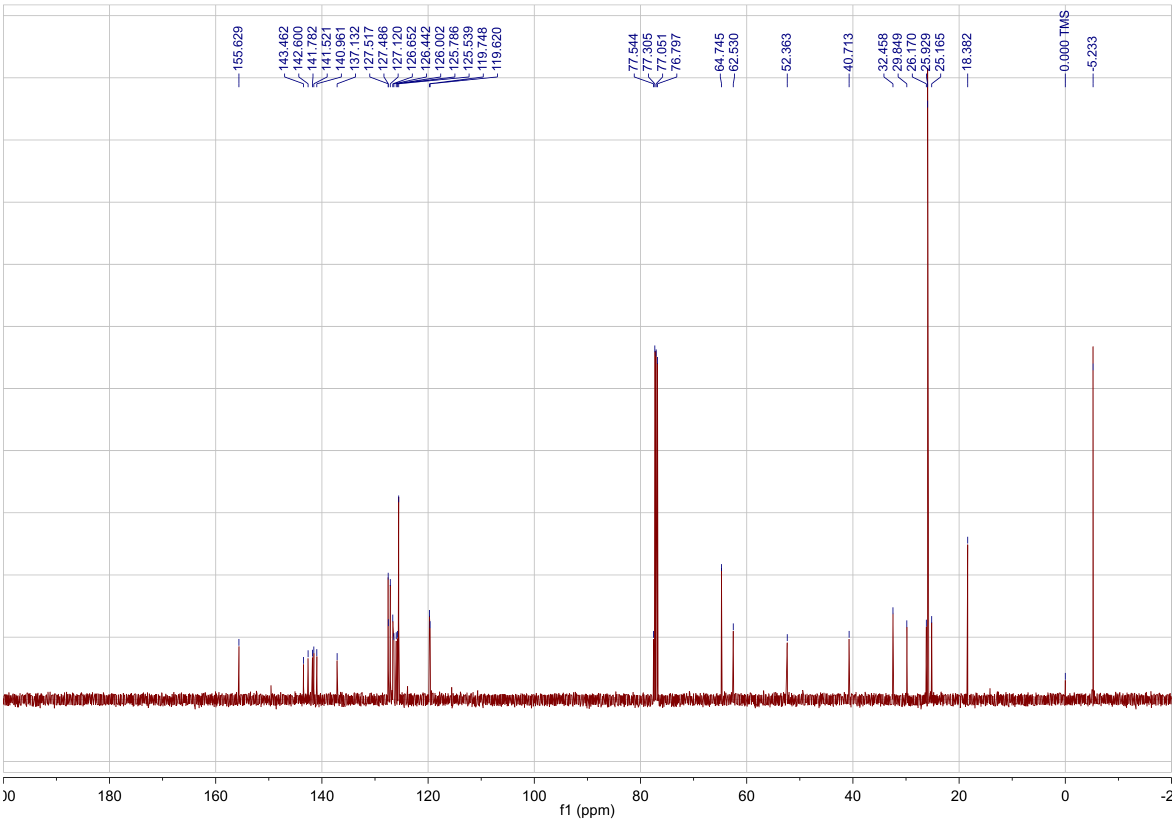

Figure S3. ^{1}H NMR and ^{13}C NMR spectra of compound **4b**

^1H NMR ($CDCl_3$)

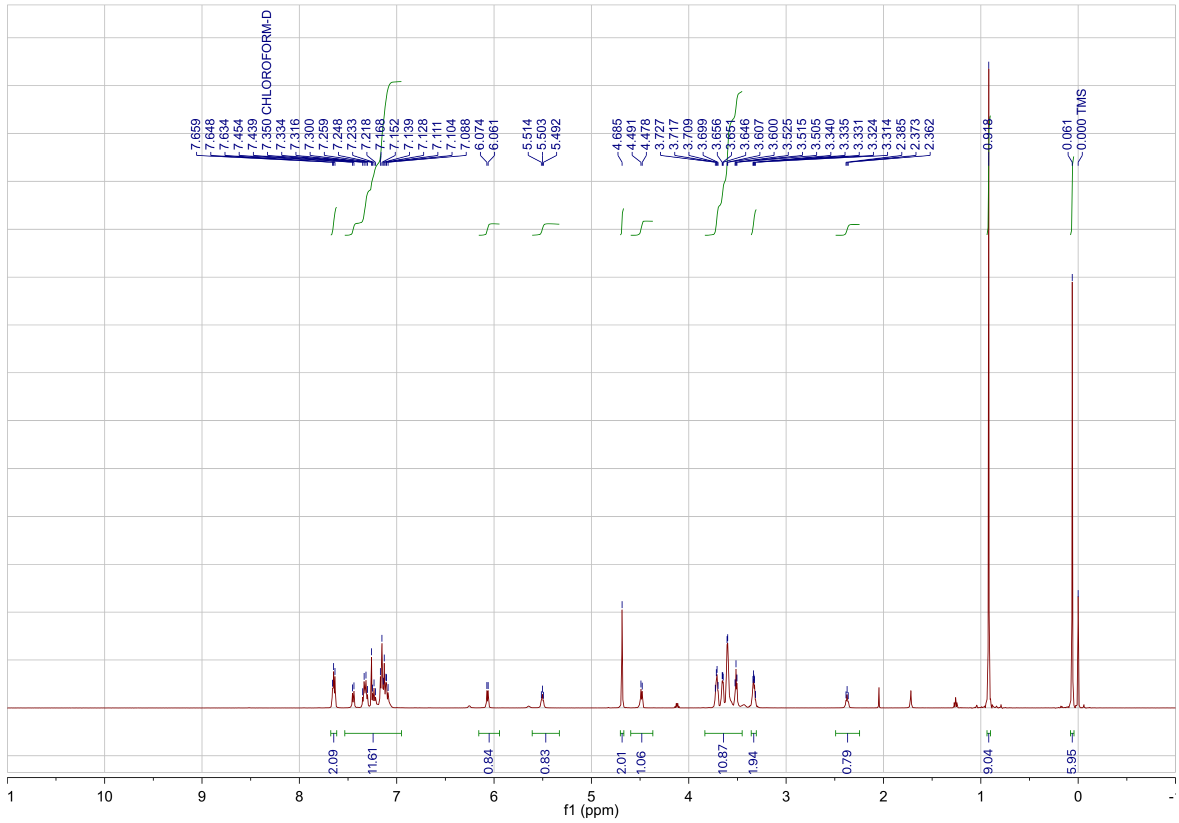

^{13}C NMR ($CDCl_3$)

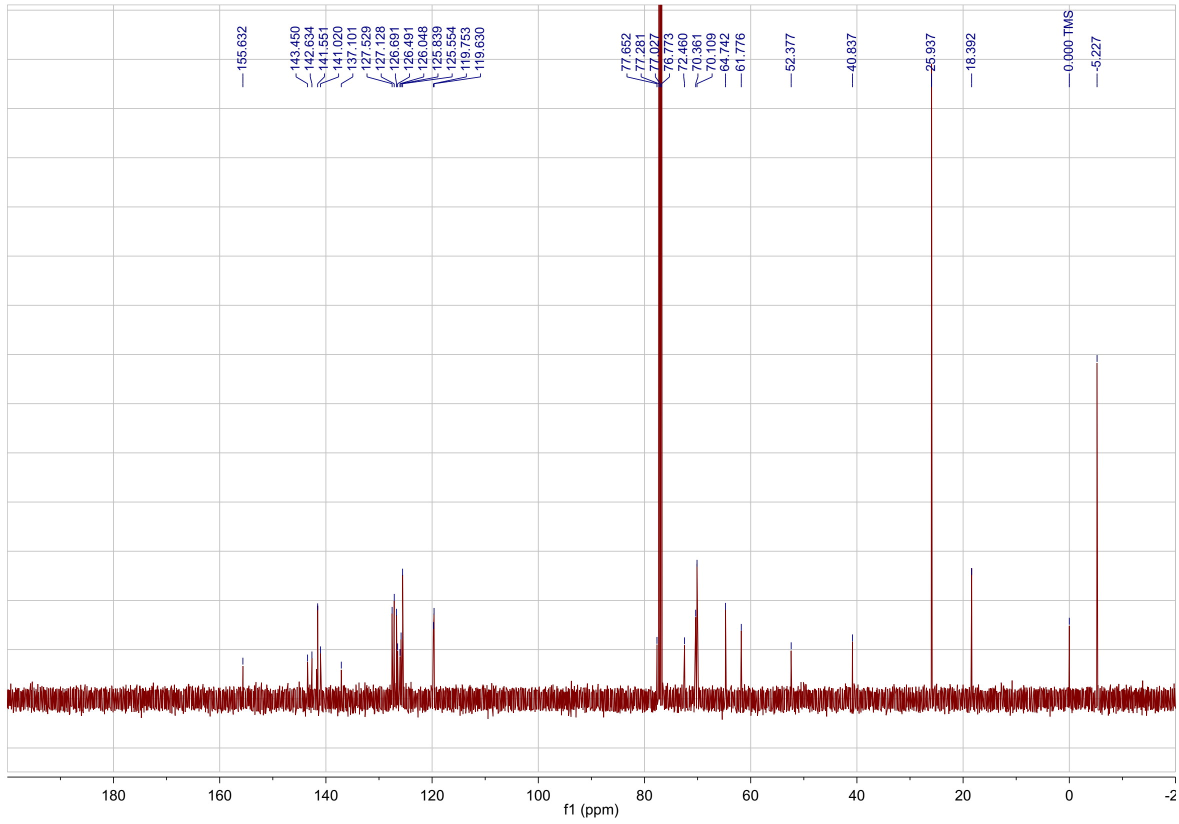

Figure S4. ^1H NMR and ^{13}C NMR spectra of compound **4c**

^{1}H NMR ($CDCl_3$)

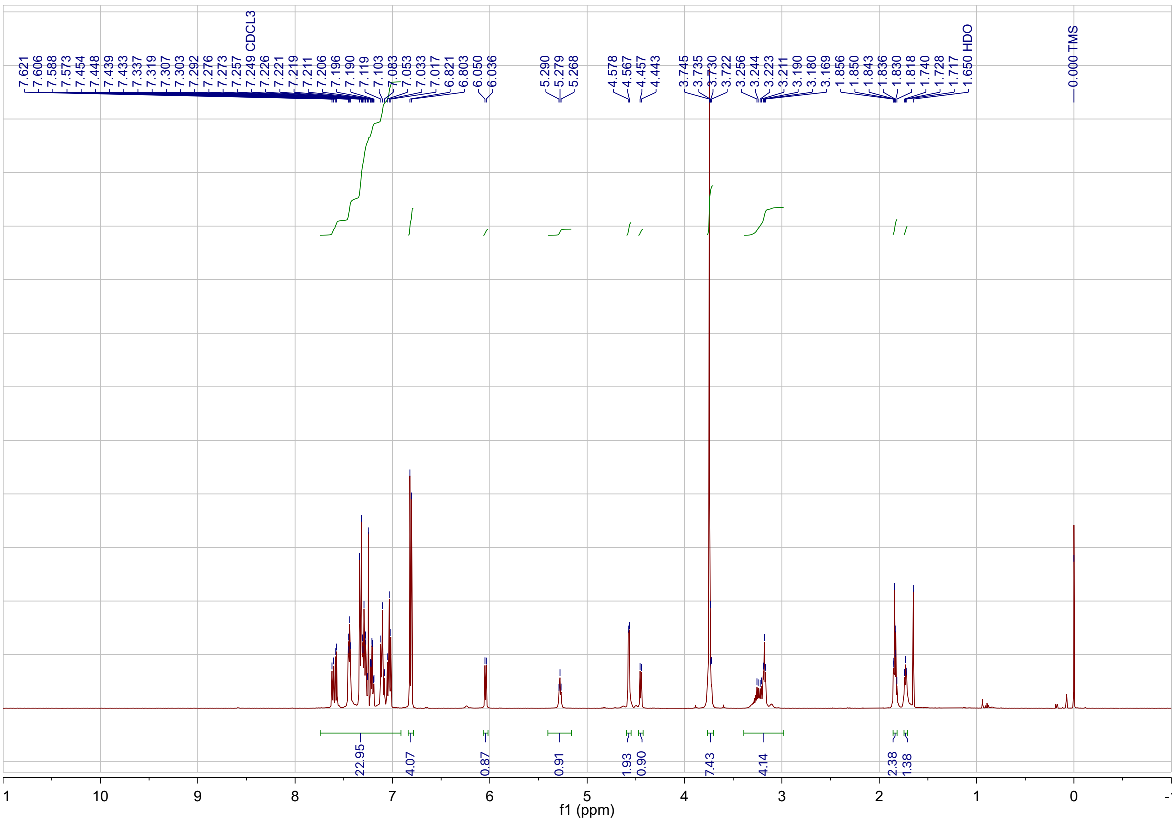

^{13}C NMR ($CDCl_3$)

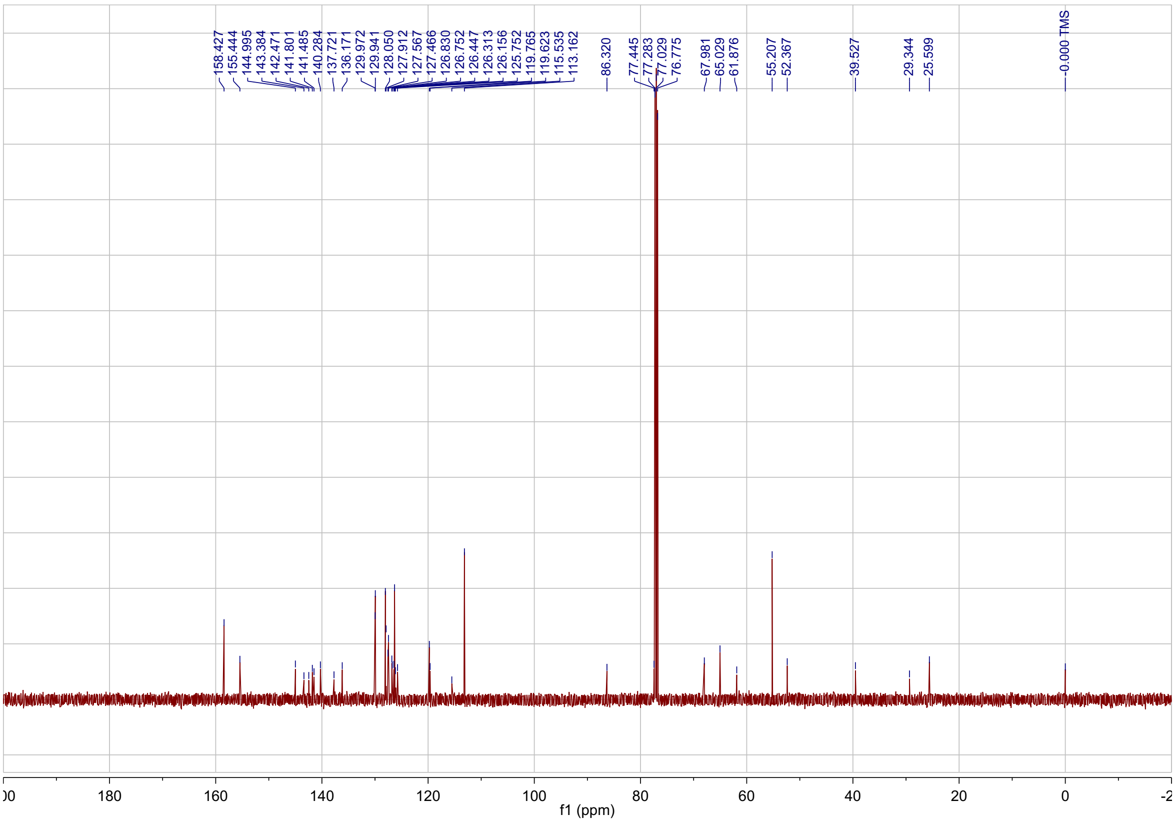

Figure S5. ^{1}H NMR and ^{13}C NMR spectra of compound **5a**

^1H NMR ($CDCl_3$)

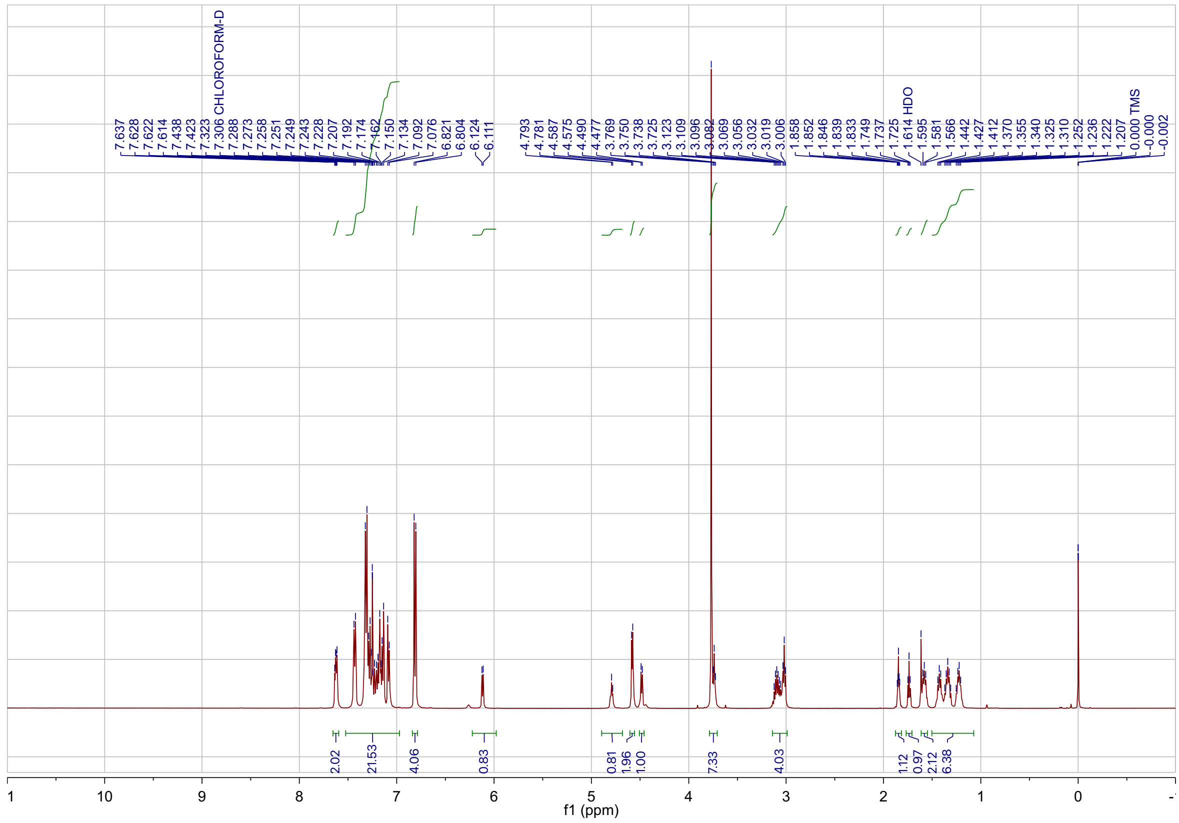

^{13}C NMR ($CDCl_3$)

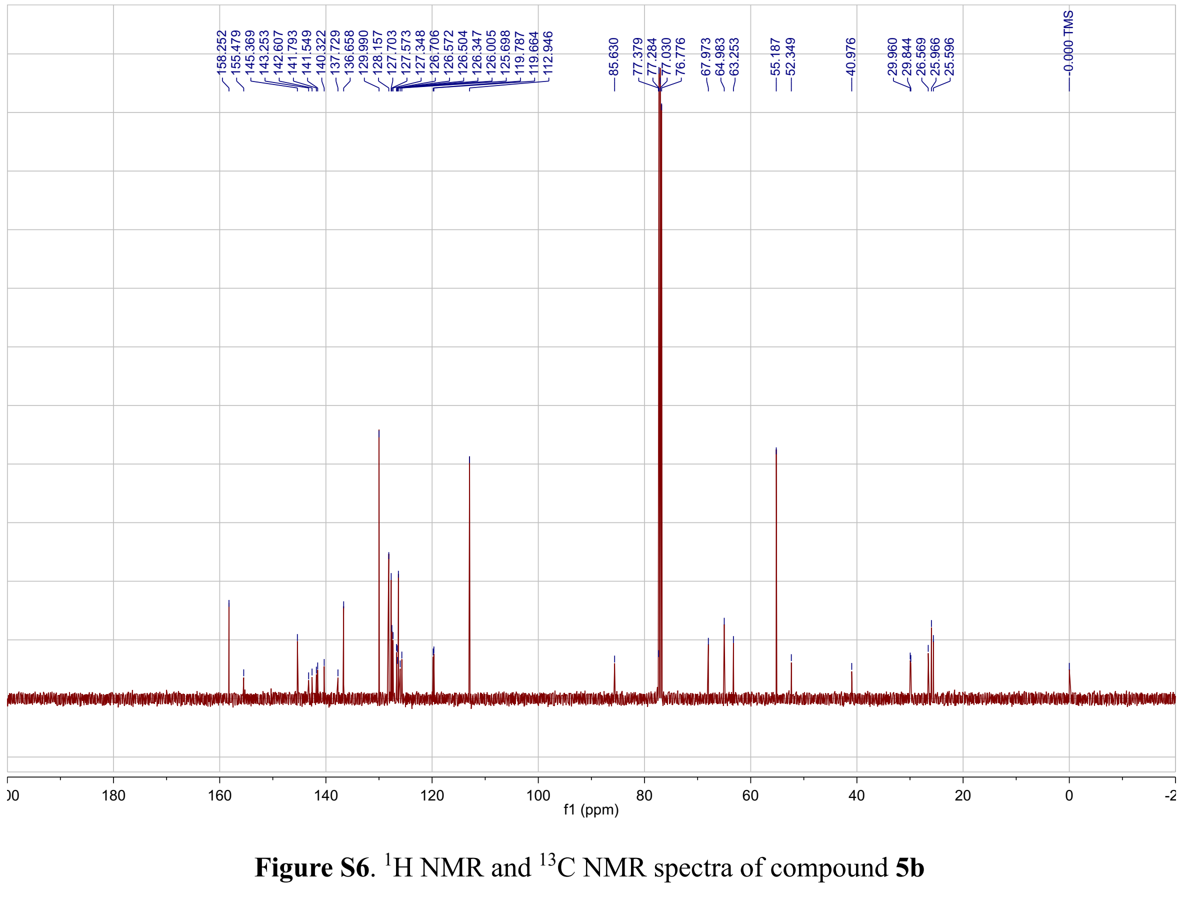

Figure S6. ^1H NMR and ^{13}C NMR spectra of compound **5b**

^{1}H NMR ($CDCl_3$)

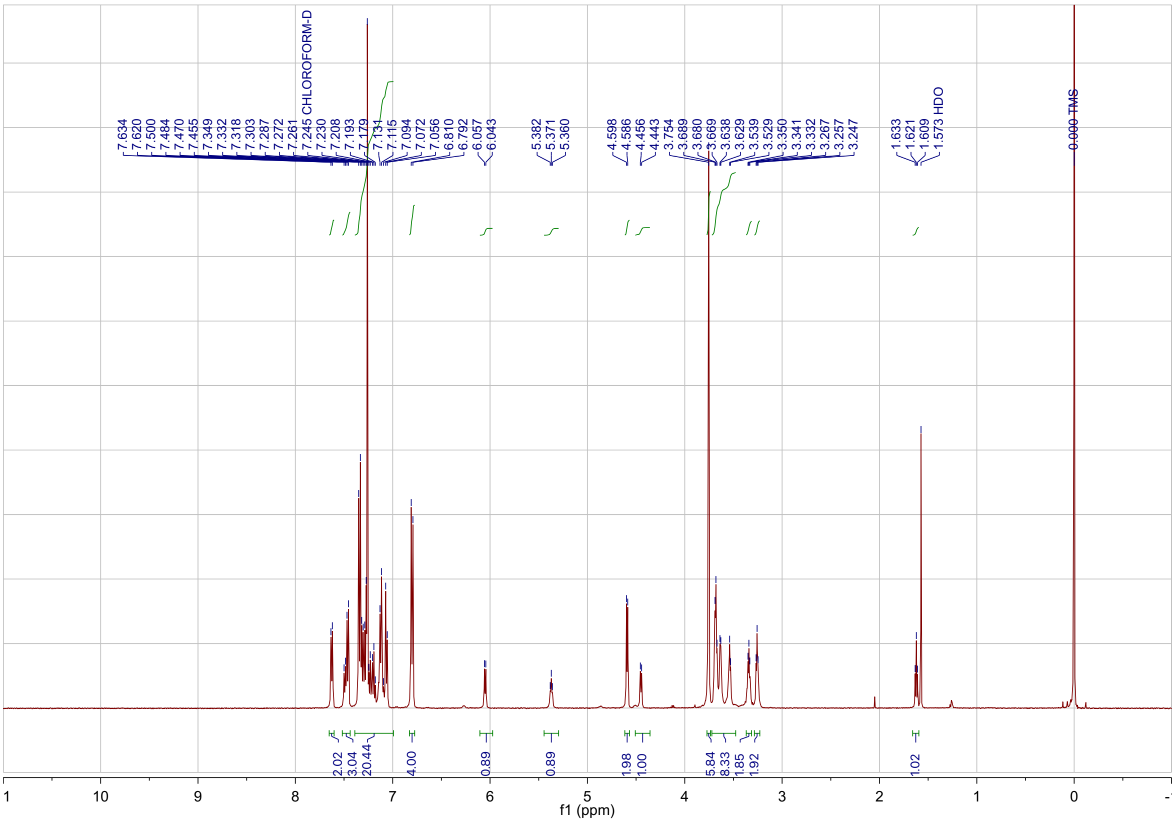

^{13}C NMR ($CDCl_3$)

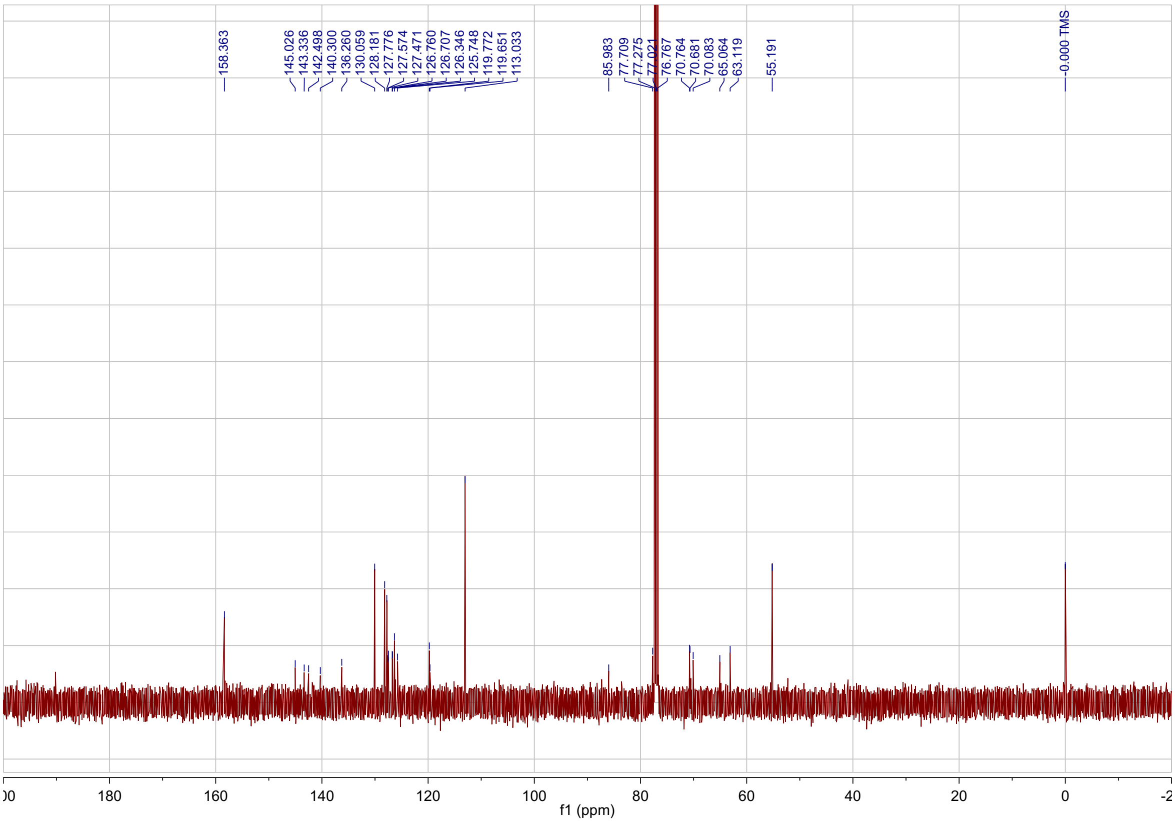

Figure S7. ^{1}H NMR and ^{13}C NMR spectra of compound **5c**

^1H NMR ($CDCl_3$)

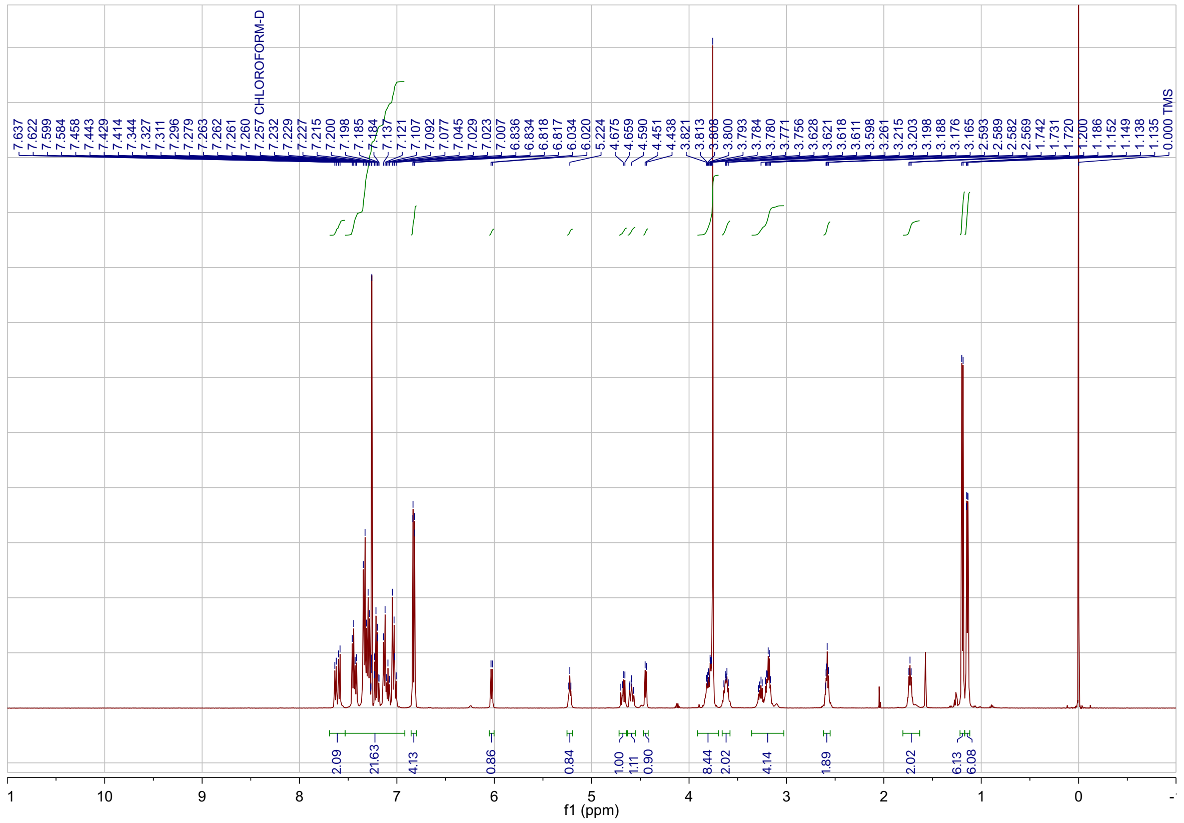

^{31}P NMR ($CDCl_3$)

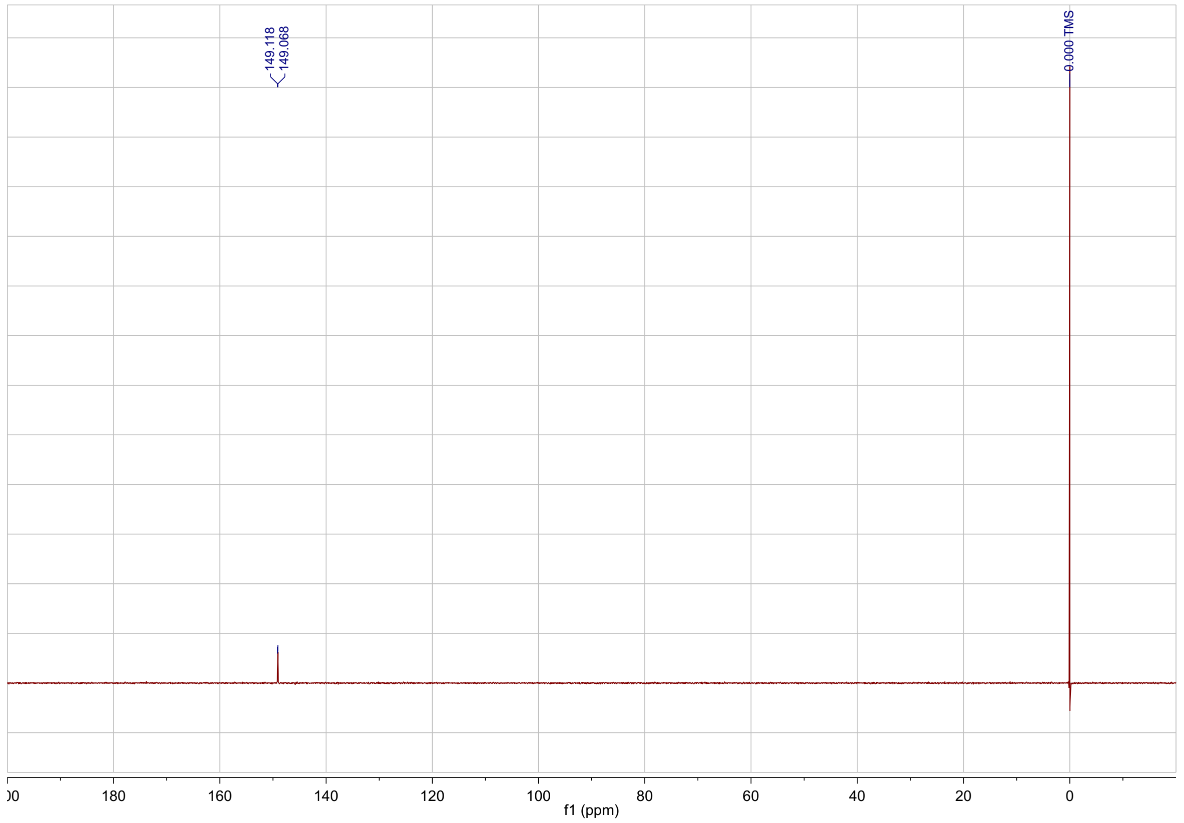

Figure S8. ^1H NMR and ^{31}P NMR spectra of compound **6a**

^{1}H NMR ($CDCl_3$)

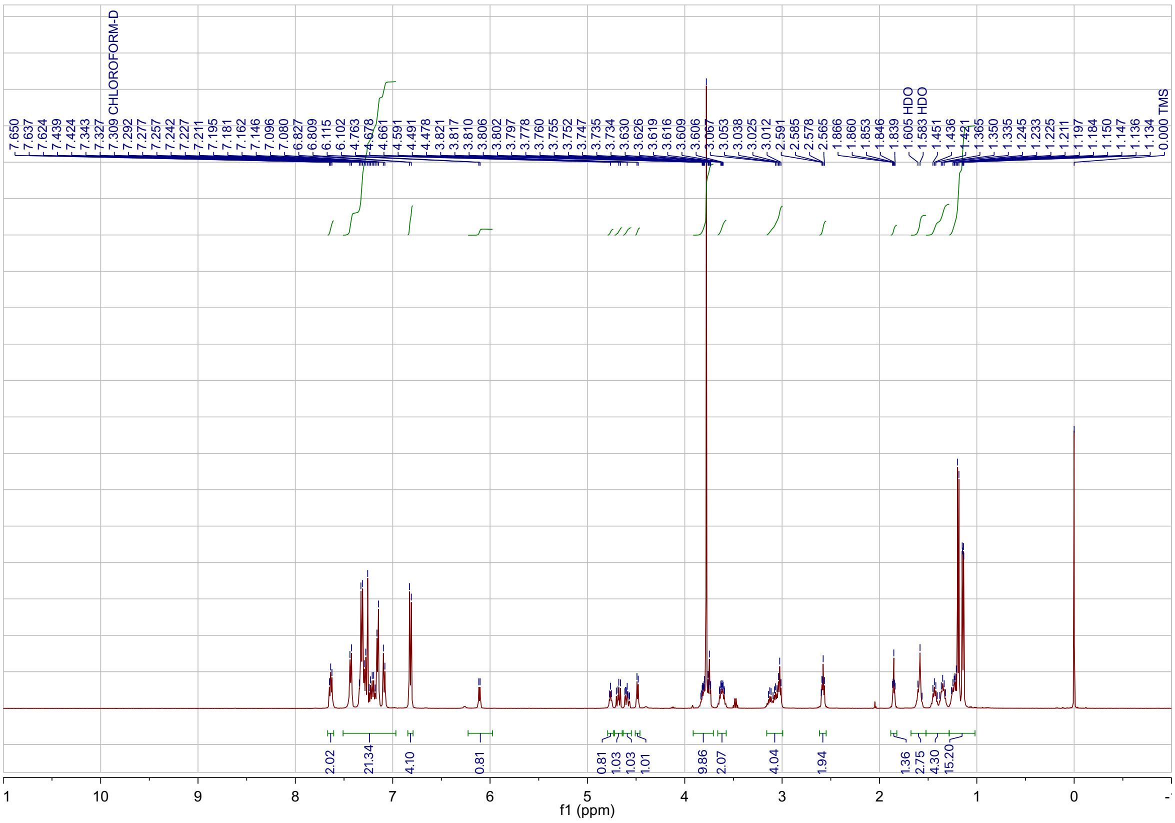

^{31}P NMR ($CDCl_3$)

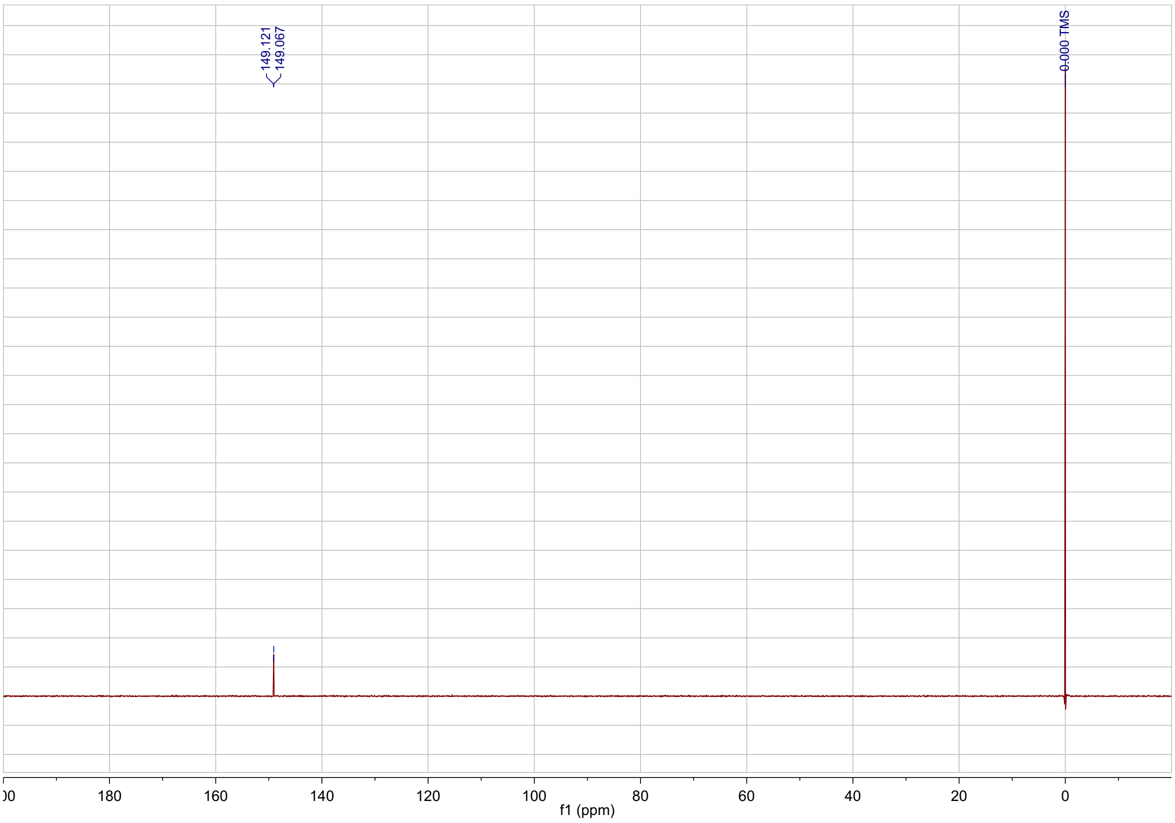

Figure S9. ^{1}H NMR and ^{31}P NMR spectra of compound **6b**

^{1}H NMR ($CDCl_3$)

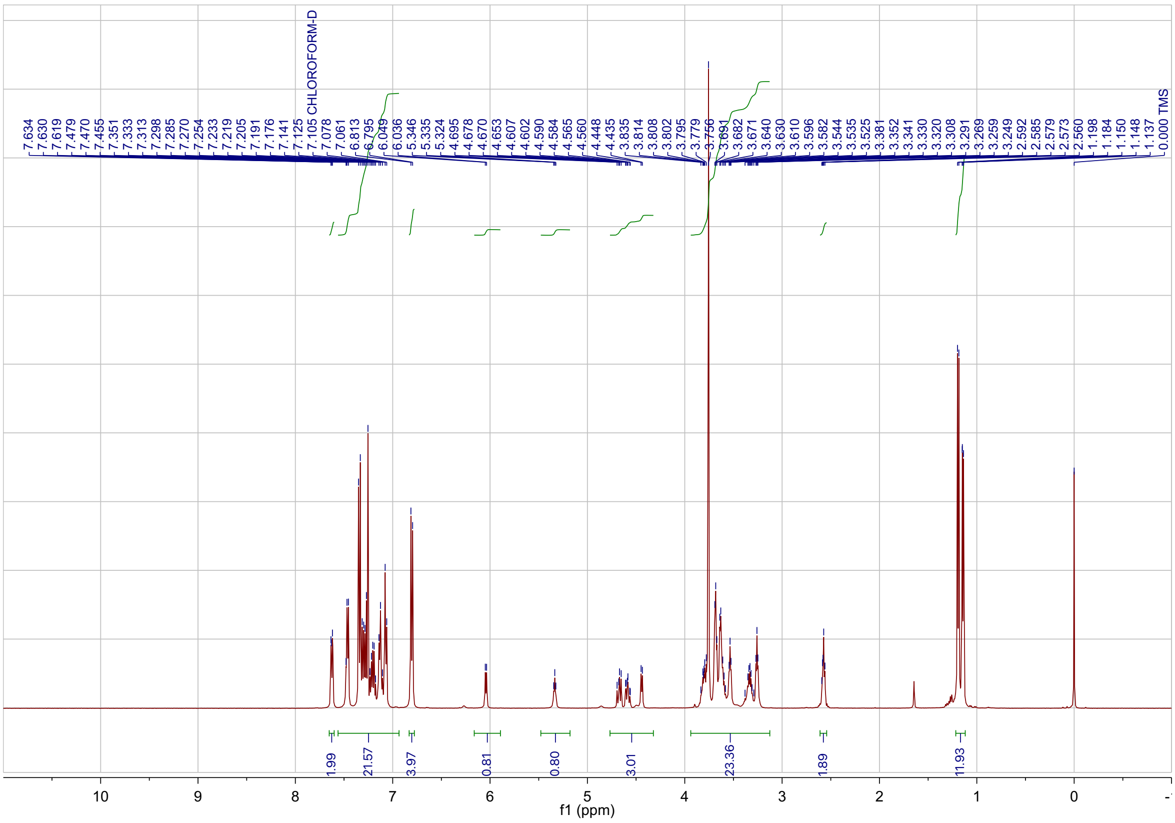

^{31}P NMR ($CDCl_3$)

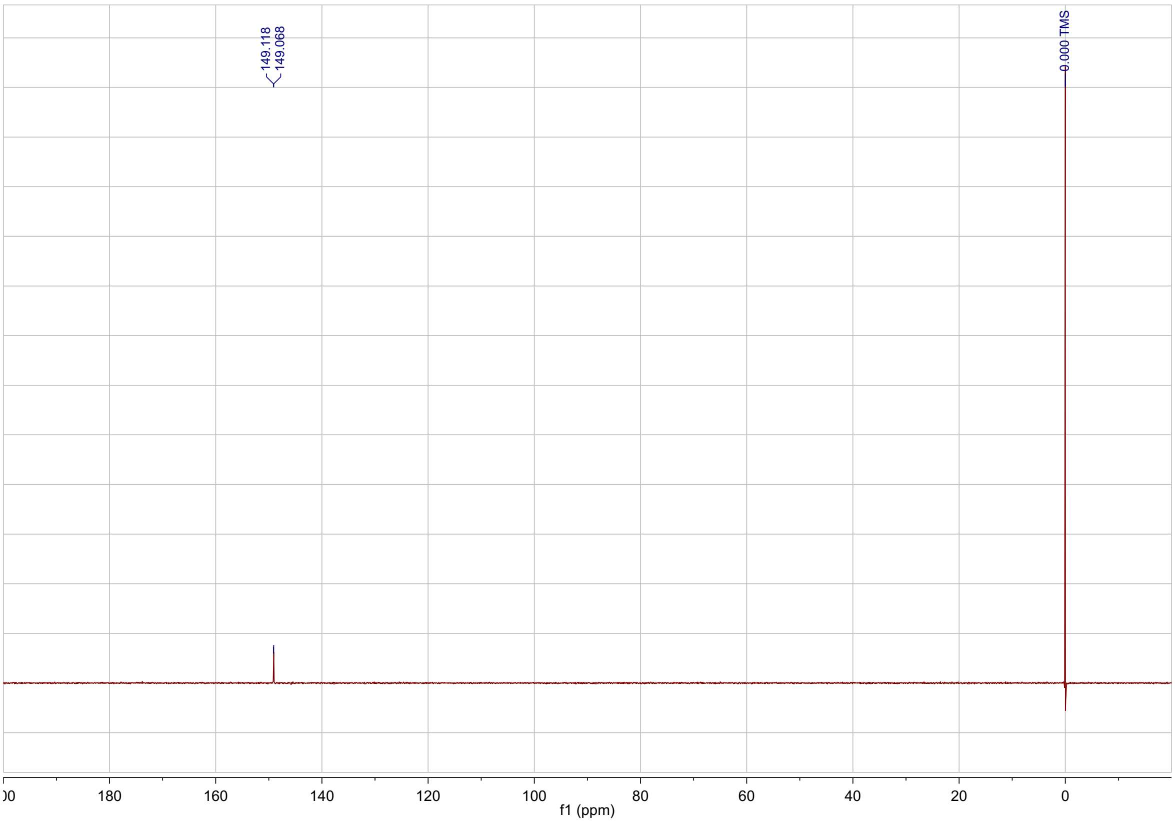

Figure S10. ^{1}H NMR and [13]P NMR spectra of compound **6c**

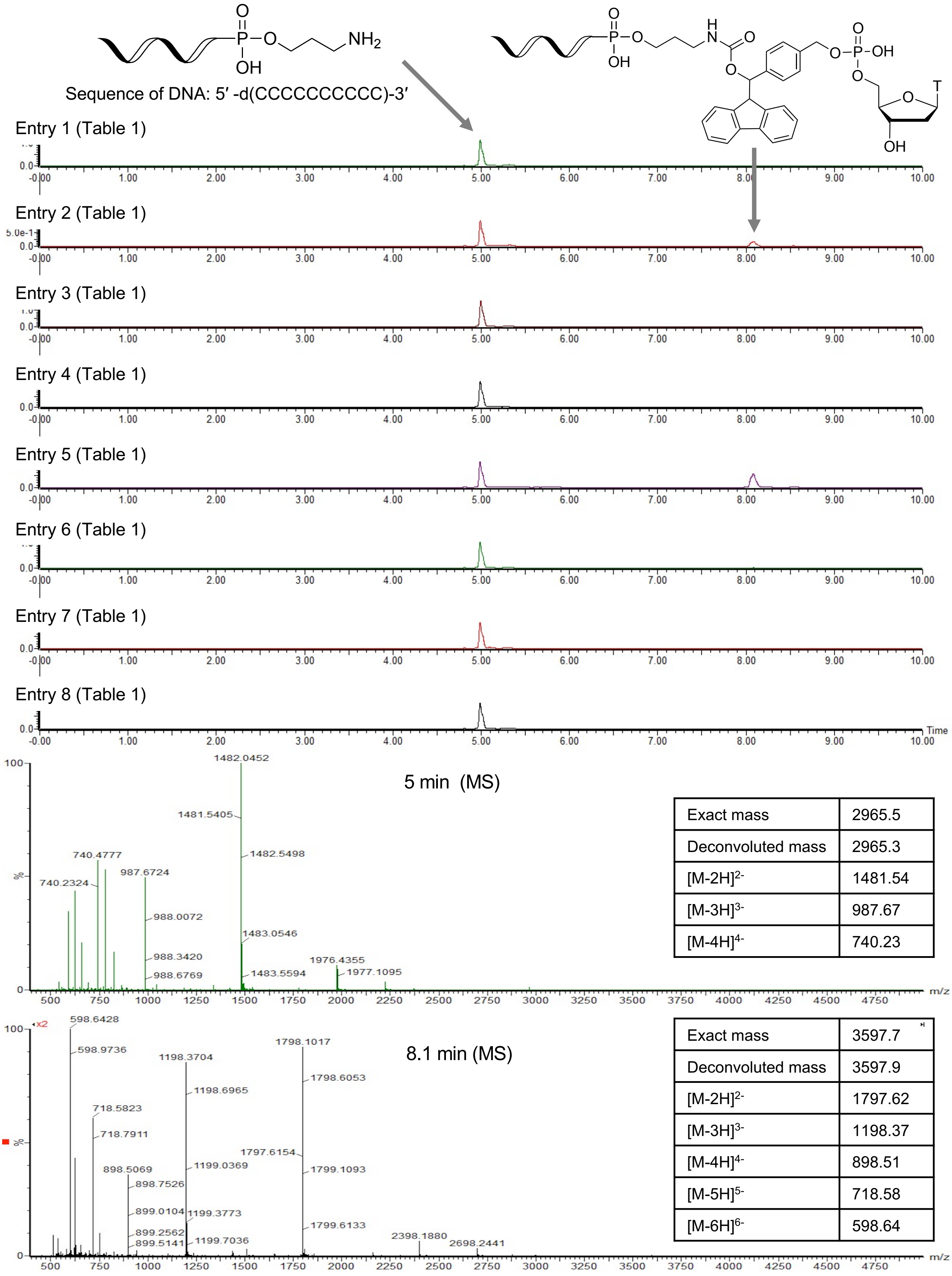

Exact mass	2965.5
Deconvoluted mass	2965.3
$[M-2H]^{2-}$	1481.54
$[M-3H]^{3-}$	987.67
$[M-4H]^{4-}$	740.23

Exact mass	3597.7
Deconvoluted mass	3597.9
$[M-2H]^{2-}$	1797.62
$[M-3H]^{3-}$	1198.37
$[M-4H]^{4-}$	898.51
$[M-5H]^{5-}$	718.58
$[M-6H]^{6-}$	598.64

Figure S11. LC/MS charts of crude C10-mer oligonucleotides

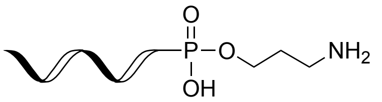

Sequence of DNA: 5′ -d(ATGCTATTCTCGA)-3′

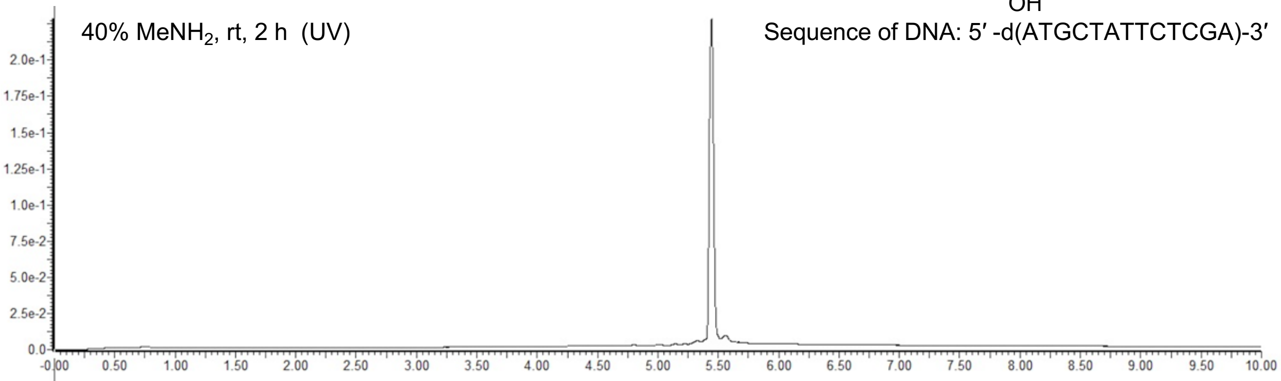

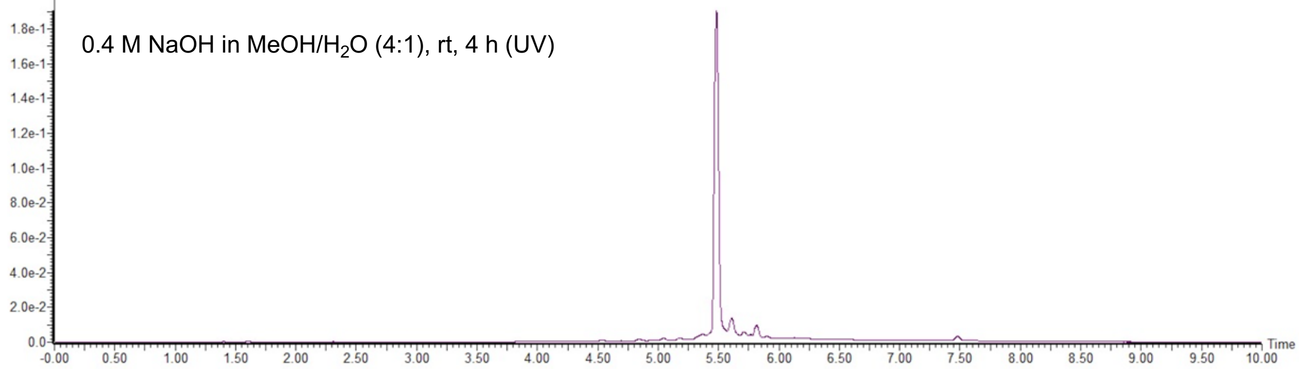

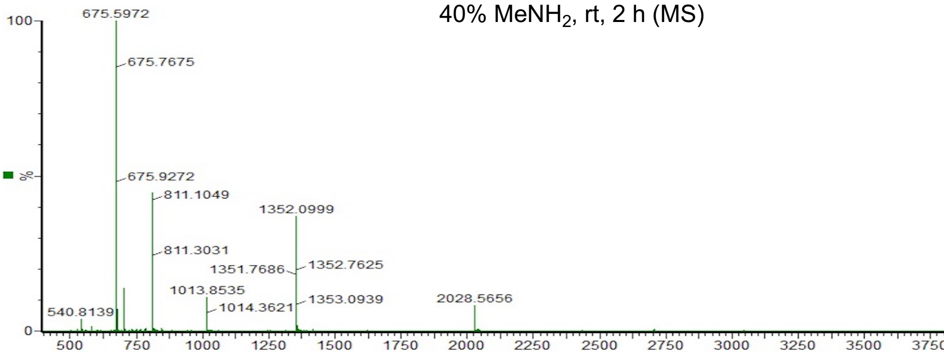

Exact mass	4059.7
Deconvoluted mass	4059.4
$[M-2H]^{2-}$	2028.57
$[M-3H]^{3-}$	1351.77
$[M-4H]^{4-}$	1013.85
$[M-5H]^{5-}$	811.11
$[M-6H]^{6-}$	675.60

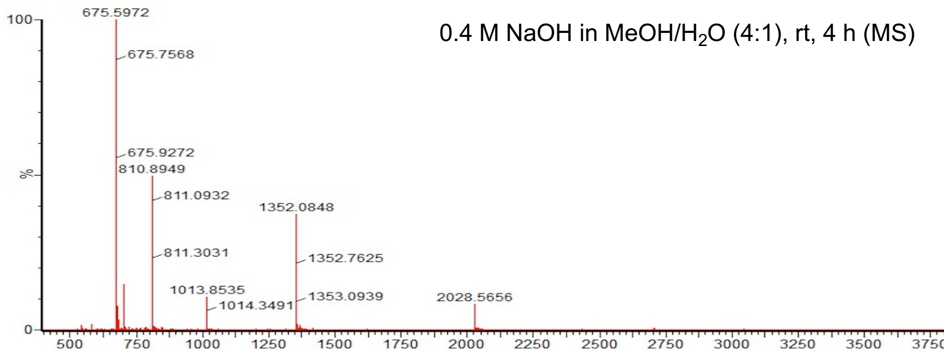

Exact mass	4059.7
Deconvoluted mass	4059.4
$[M-2H]^{2-}$	2028.57
$[M-3H]^{3-}$	1352.08
$[M-4H]^{4-}$	1013.85
$[M-5H]^{5-}$	810.89
$[M-6H]^{6-}$	675.60

Figure S12. LC/MS charts of crude 13-mer oligonucleotides with a 3′-aminopropyl linker

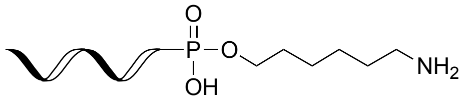

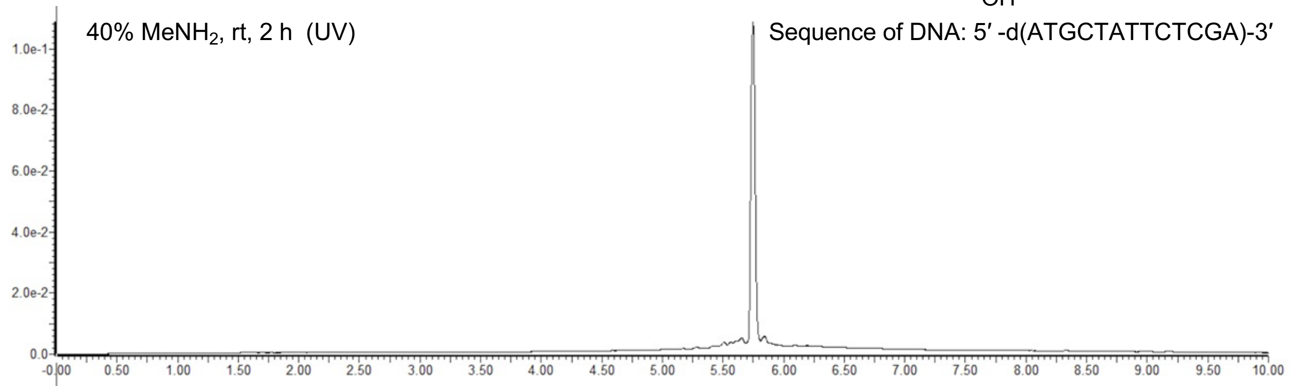

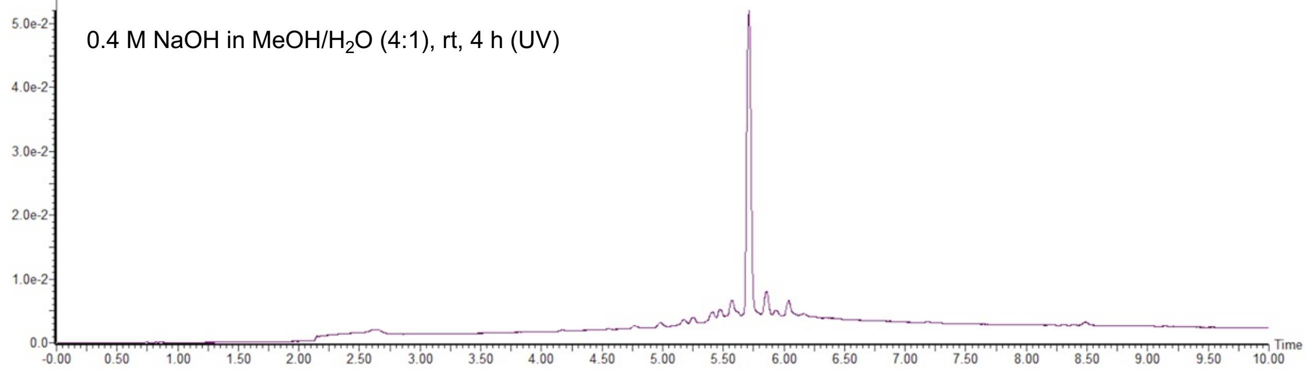

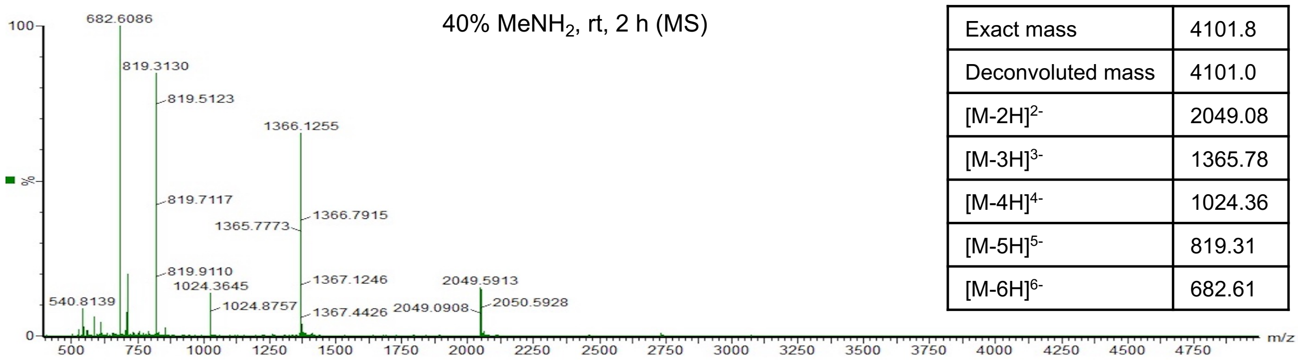

Exact mass	4101.8
Deconvoluted mass	4101.0
$[M-2H]^{2-}$	2049.08
$[M-3H]^{3-}$	1365.78
$[M-4H]^{4-}$	1024.36
$[M-5H]^{5-}$	819.31
$[M-6H]^{6-}$	682.61

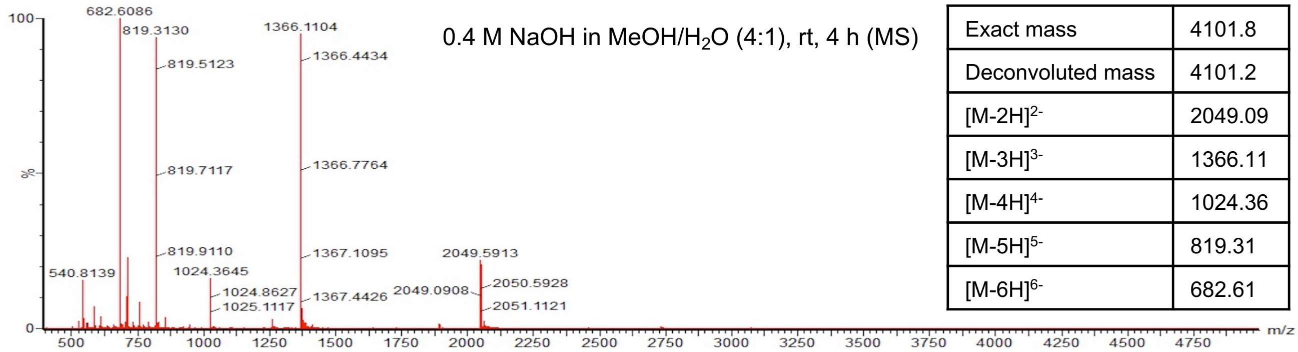

Exact mass	4101.8
Deconvoluted mass	4101.2
$[M-2H]^{2-}$	2049.09
$[M-3H]^{3-}$	1366.11
$[M-4H]^{4-}$	1024.36
$[M-5H]^{5-}$	819.31
$[M-6H]^{6-}$	682.61

Figure S13. LC/MS charts of crude 13-mer oligonucleotides with a 3′-aminohexyl linker

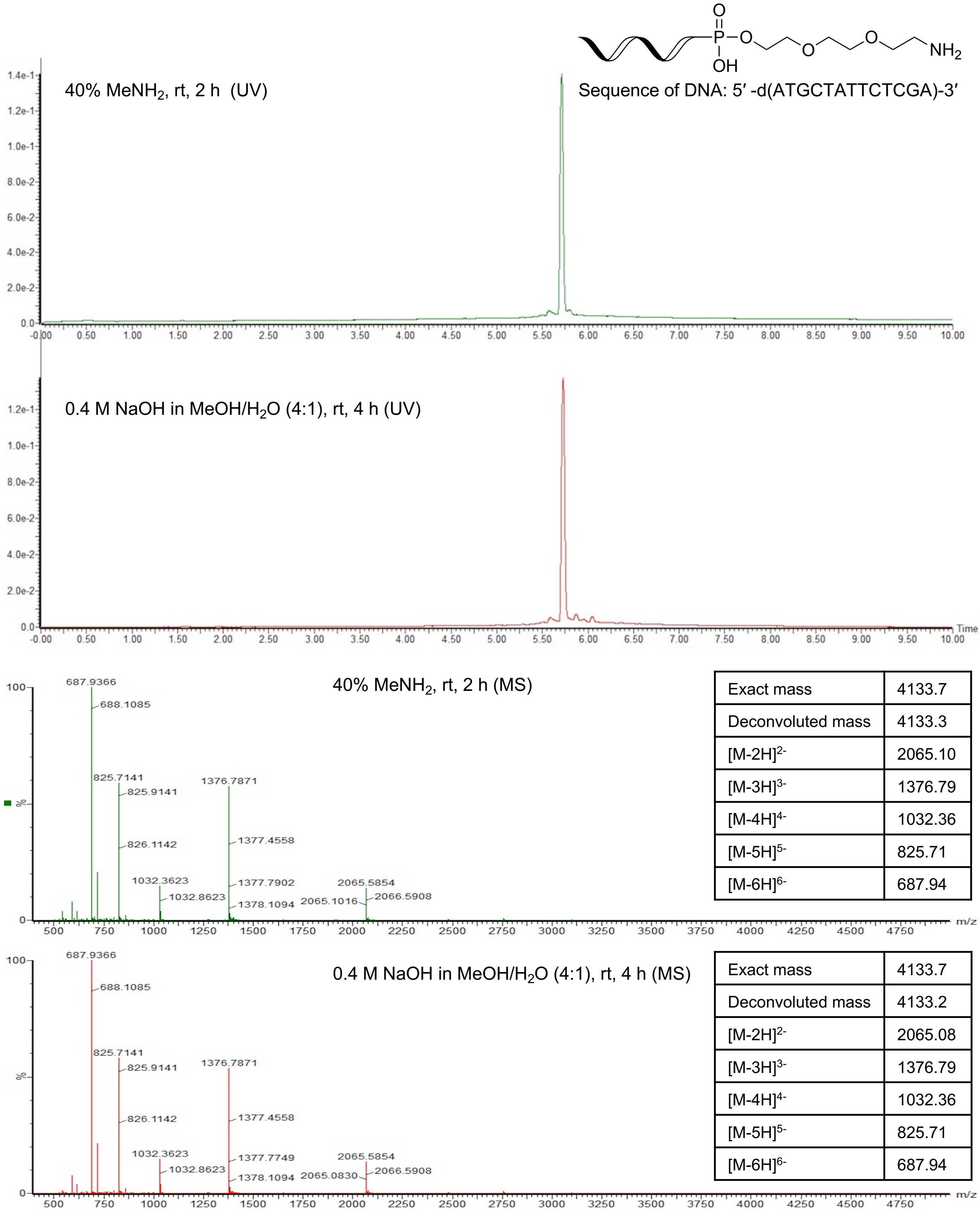

Exact mass	4133.7
Deconvoluted mass	4133.3
$[M-2H]^{2-}$	2065.10
$[M-3H]^{3-}$	1376.79
$[M-4H]^{4-}$	1032.36
$[M-5H]^{5-}$	825.71
$[M-6H]^{6-}$	687.94

Exact mass	4133.7
Deconvoluted mass	4133.2
$[M-2H]^{2-}$	2065.08
$[M-3H]^{3-}$	1376.79
$[M-4H]^{4-}$	1032.36
$[M-5H]^{5-}$	825.71
$[M-6H]^{6-}$	687.94

Figure S14. LC/MS charts of crude 13-mer oligonucleotides with a 3′-amino-PEG2 linker

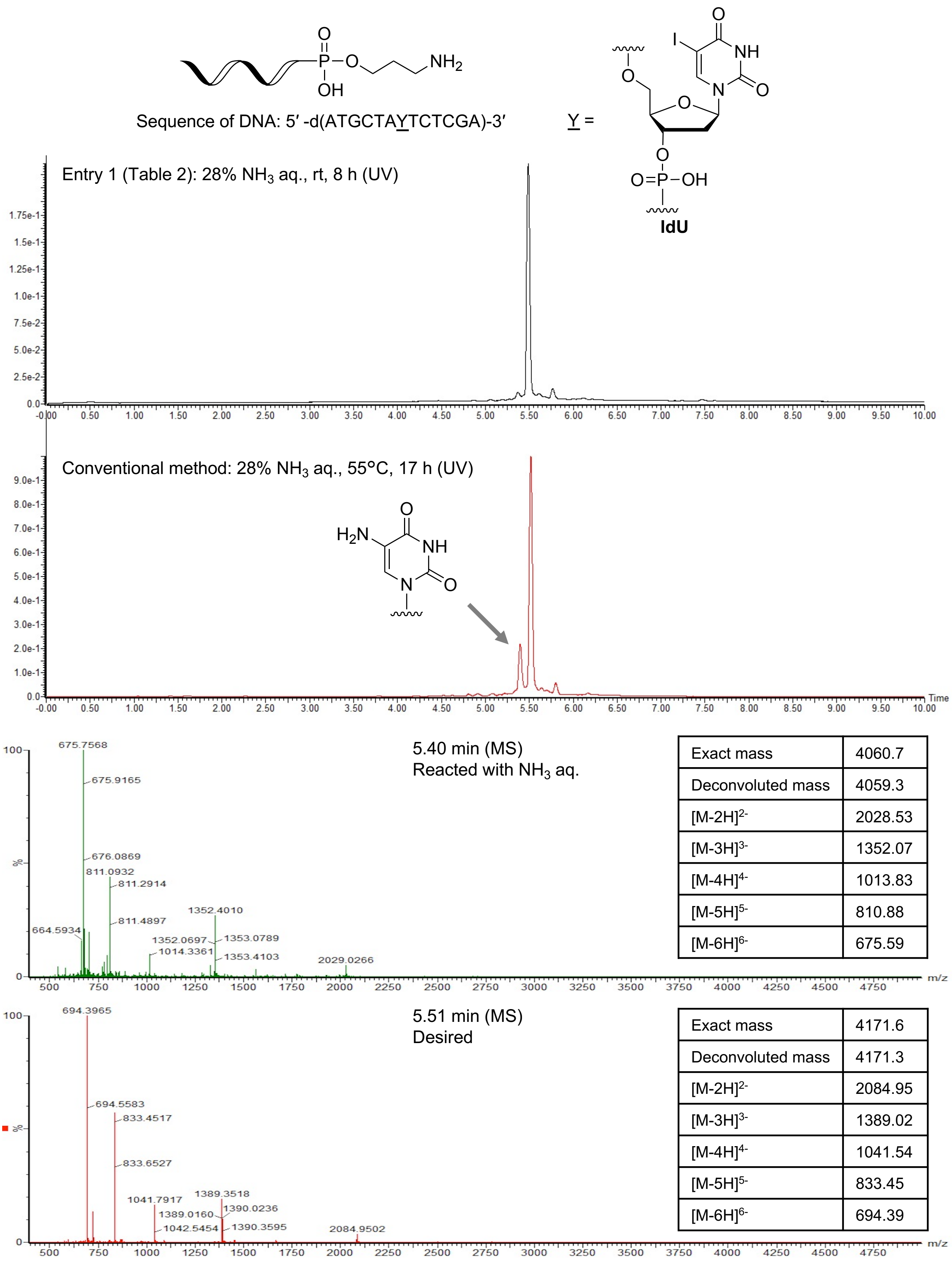

Exact mass	4060.7
Deconvoluted mass	4059.3
$[M-2H]^{2-}$	2028.53
$[M-3H]^{3-}$	1352.07
$[M-4H]^{4-}$	1013.83
$[M-5H]^{5-}$	810.88
$[M-6H]^{6-}$	675.59

Exact mass	4171.6
Deconvoluted mass	4171.3
$[M-2H]^{2-}$	2084.95
$[M-3H]^{3-}$	1389.02
$[M-4H]^{4-}$	1041.54
$[M-5H]^{5-}$	833.45
$[M-6H]^{6-}$	694.39

Figure S15. LC/MS charts of crude IdU-modified oligonucleotides with a 3′-aminopropyl linker

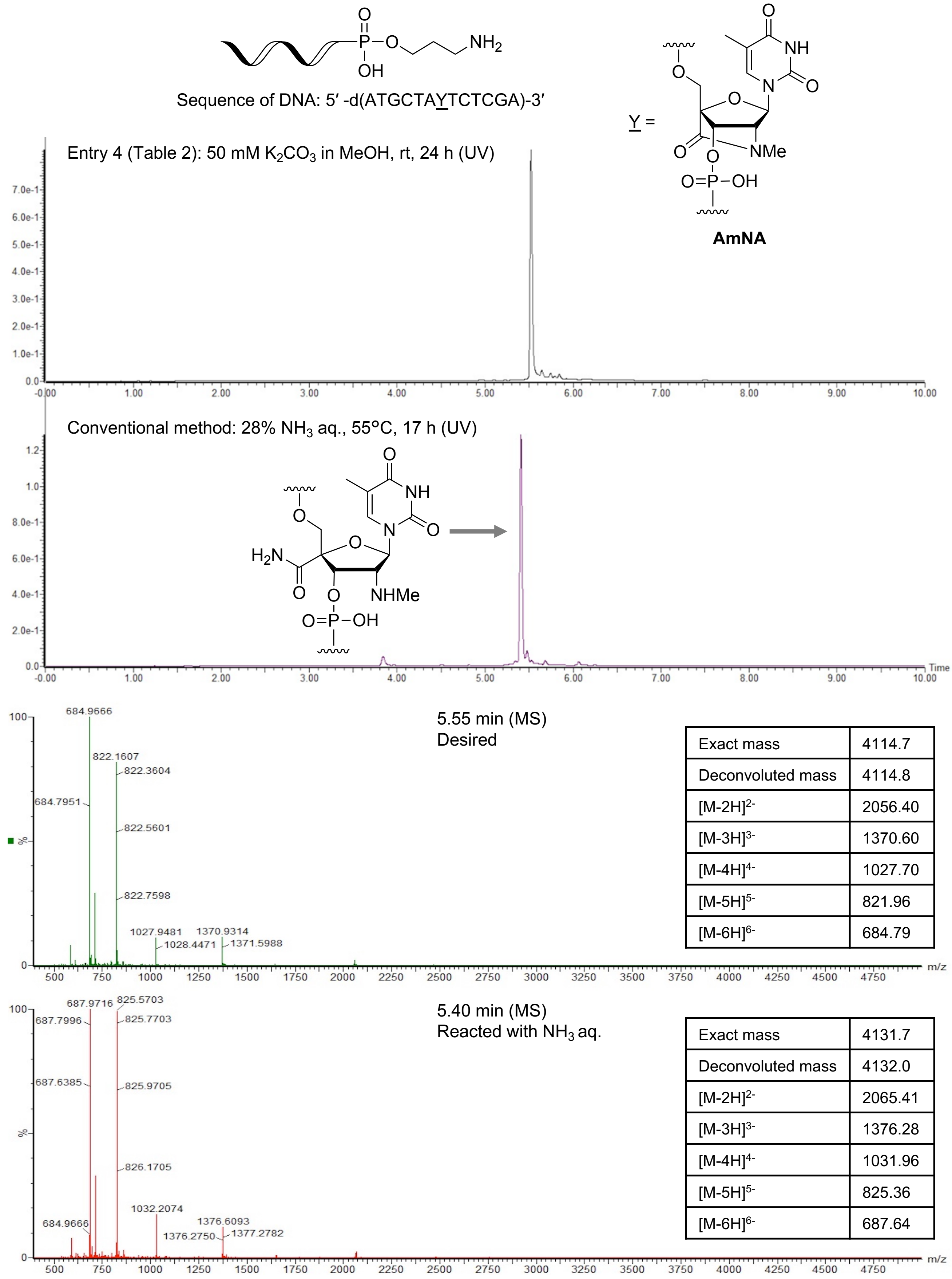

Exact mass	4114.7
Deconvoluted mass	4114.8
$[M-2H]^{2-}$	2056.40
$[M-3H]^{3-}$	1370.60
$[M-4H]^{4-}$	1027.70
$[M-5H]^{5-}$	821.96
$[M-6H]^{6-}$	684.79

Exact mass	4131.7
Deconvoluted mass	4132.0
$[M-2H]^{2-}$	2065.41
$[M-3H]^{3-}$	1376.28
$[M-4H]^{4-}$	1031.96
$[M-5H]^{5-}$	825.36
$[M-6H]^{6-}$	687.64

Figure S16. LC/MS charts of crude AmNA-modified oligonucleotides with a 3′-aminopropyl linker

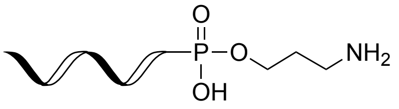

Sequence of DNA: 5′ -d(ATGCTAYTCTCGA)-3′

Y =

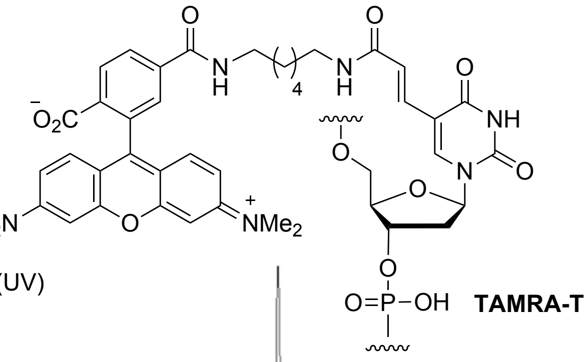

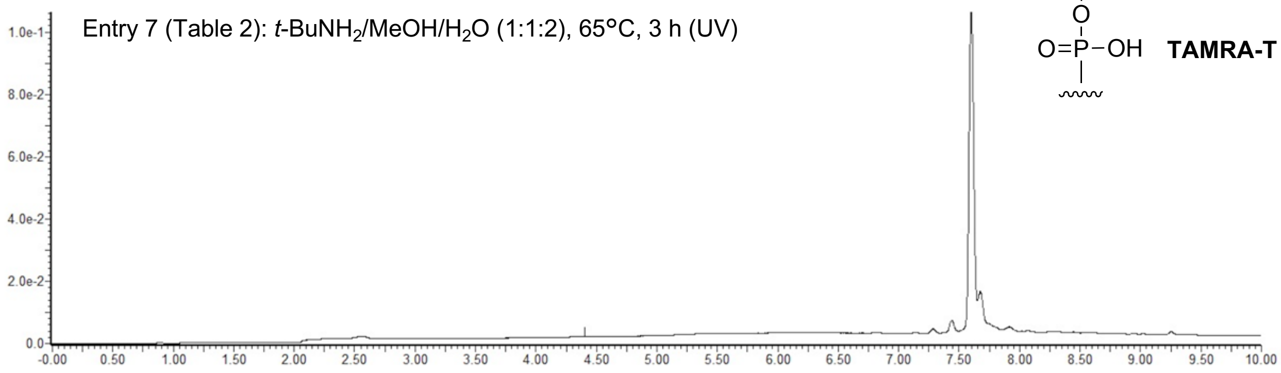

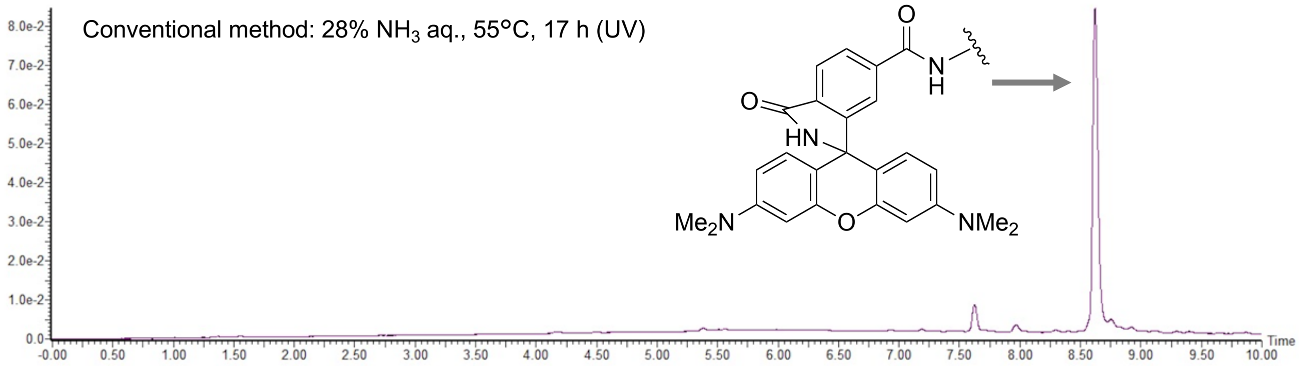

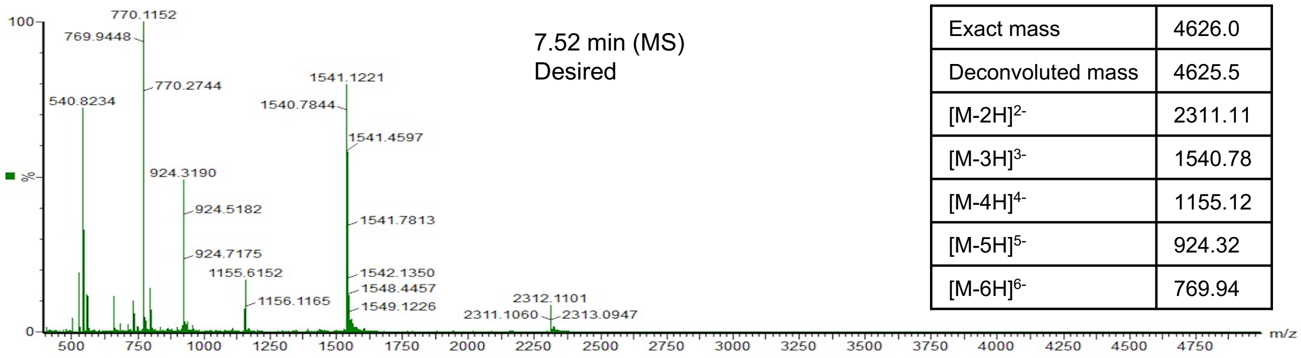

Exact mass	4626.0
Deconvoluted mass	4625.5
$[M-2H]^{2-}$	2311.11
$[M-3H]^{3-}$	1540.78
$[M-4H]^{4-}$	1155.12
$[M-5H]^{5-}$	924.32
$[M-6H]^{6-}$	769.94

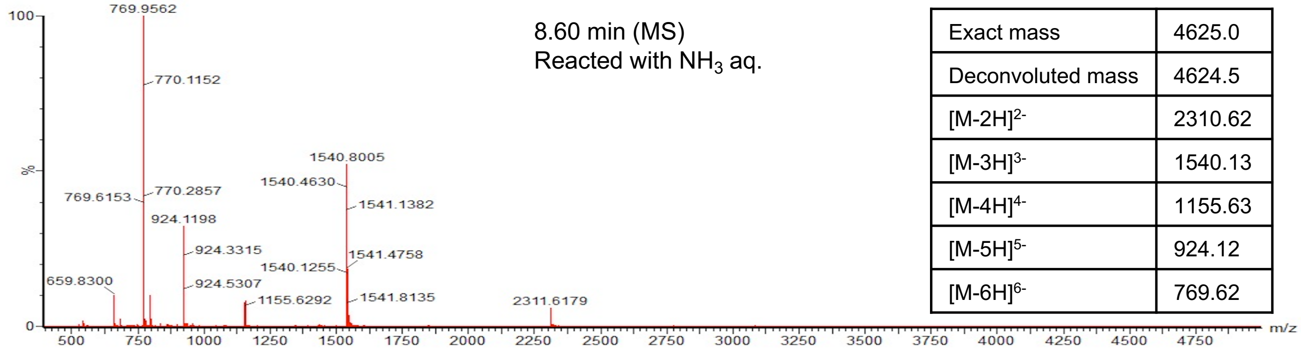

Exact mass	4625.0
Deconvoluted mass	4624.5
$[M-2H]^{2-}$	2310.62
$[M-3H]^{3-}$	1540.13
$[M-4H]^{4-}$	1155.63
$[M-5H]^{5-}$	924.12
$[M-6H]^{6-}$	769.62

Figure S17. LC/MS charts of crude TAMRA-T-modified oligonucleotides with a 3′-aminopropyl linker

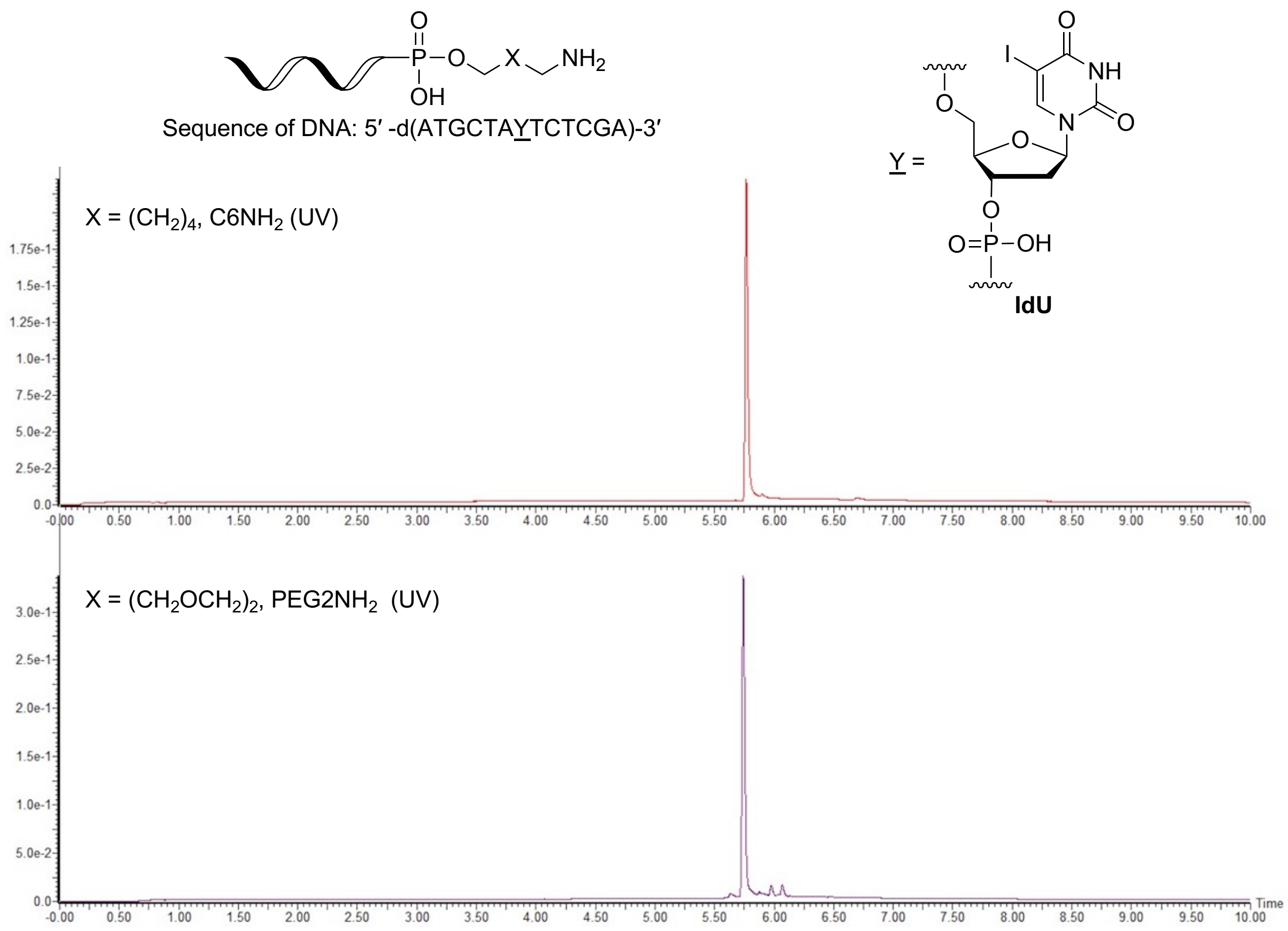

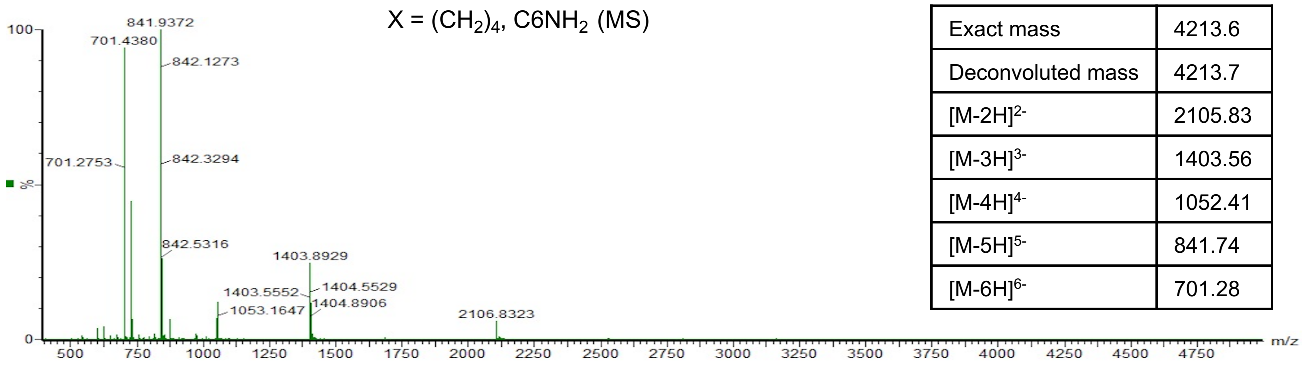

Exact mass	4213.6
Deconvoluted mass	4213.7
$[M-2H]^{2-}$	2105.83
$[M-3H]^{3-}$	1403.56
$[M-4H]^{4-}$	1052.41
$[M-5H]^{5-}$	841.74
$[M-6H]^{6-}$	701.28

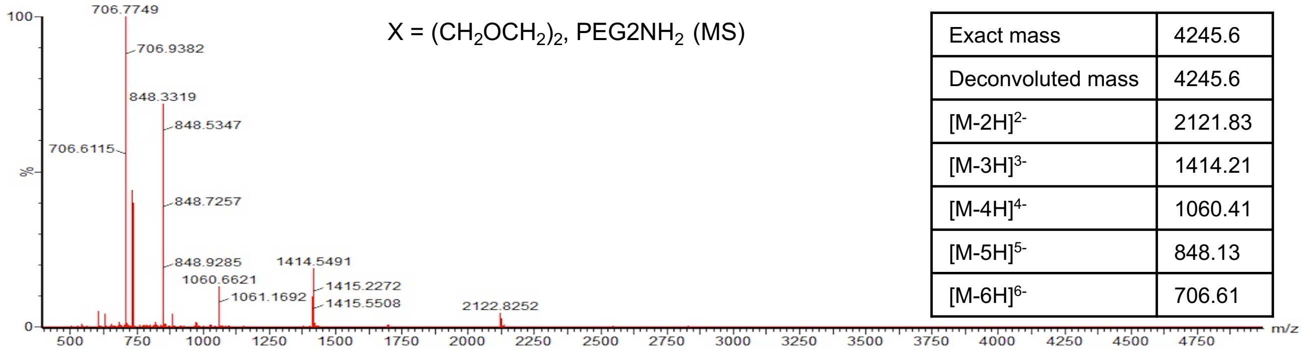

Exact mass	4245.6
Deconvoluted mass	4245.6
$[M-2H]^{2-}$	2121.83
$[M-3H]^{3-}$	1414.21
$[M-4H]^{4-}$	1060.41
$[M-5H]^{5-}$	848.13
$[M-6H]^{6-}$	706.61

Figure S18. LC/MS charts of crude IdU-modified oligonucleotides with a 3′-aminohexyl and amino-PEG2 linker

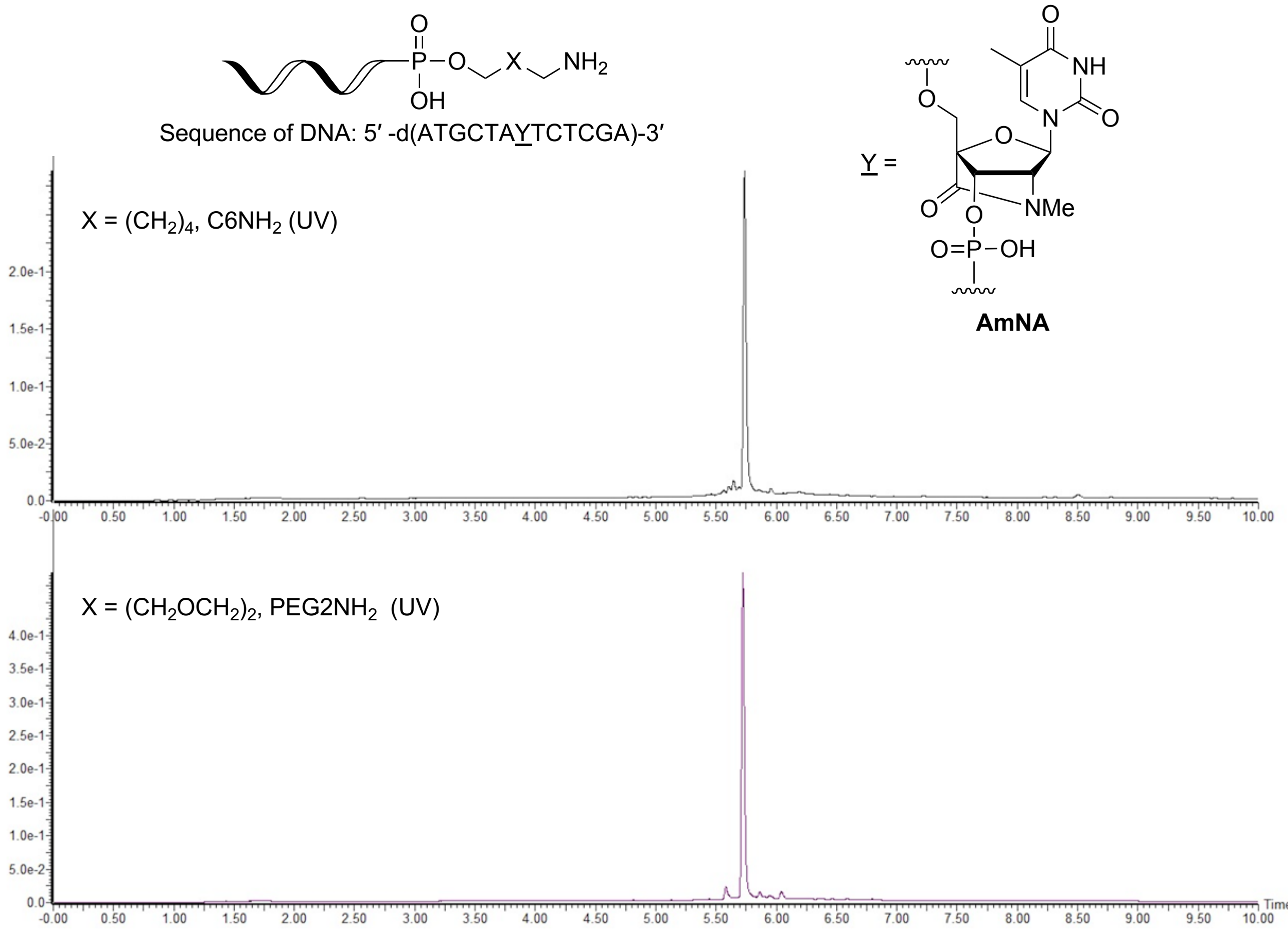

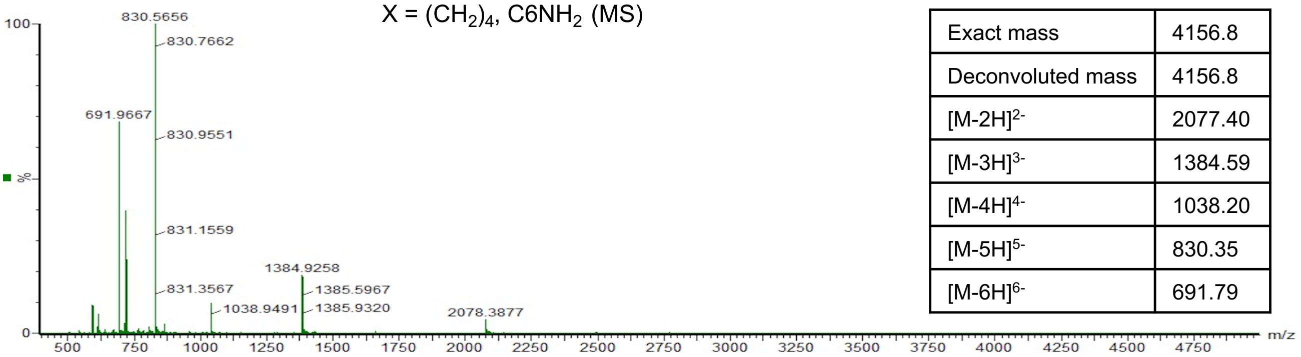

Exact mass	4156.8
Deconvoluted mass	4156.8
$[M-2H]^{2-}$	2077.40
$[M-3H]^{3-}$	1384.59
$[M-4H]^{4-}$	1038.20
$[M-5H]^{5-}$	830.35
$[M-6H]^{6-}$	691.79

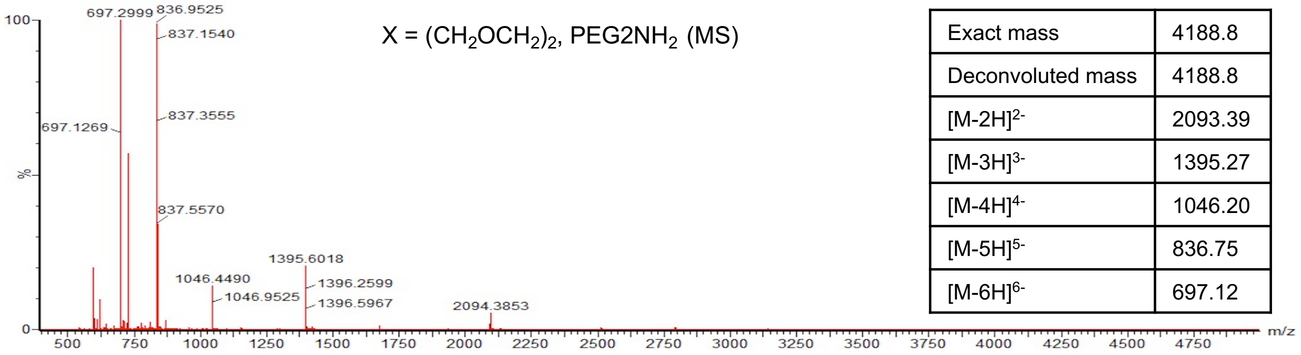

Exact mass	4188.8
Deconvoluted mass	4188.8
$[M-2H]^{2-}$	2093.39
$[M-3H]^{3-}$	1395.27
$[M-4H]^{4-}$	1046.20
$[M-5H]^{5-}$	836.75
$[M-6H]^{6-}$	697.12

Figure S19. LC/MS charts of crude AmNA-modified oligonucleotides with a 3′-aminohexyl and amino-PEG2 linker

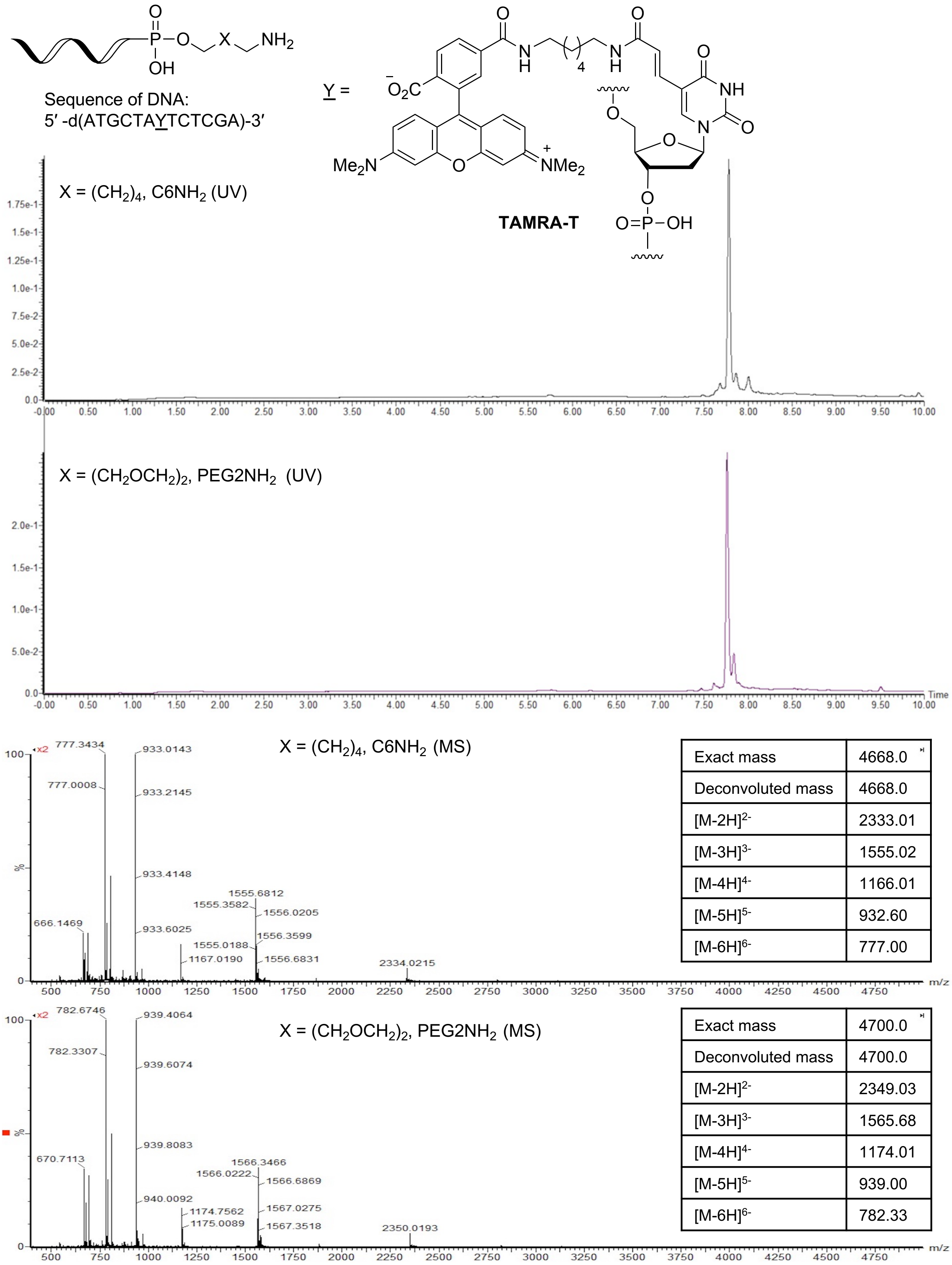

Exact mass	4668.0
Deconvoluted mass	4668.0
$[M-2H]^{2-}$	2333.01
$[M-3H]^{3-}$	1555.02
$[M-4H]^{4-}$	1166.01
$[M-5H]^{5-}$	932.60
$[M-6H]^{6-}$	777.00

Exact mass	4700.0
Deconvoluted mass	4700.0
$[M-2H]^{2-}$	2349.03
$[M-3H]^{3-}$	1565.68
$[M-4H]^{4-}$	1174.01
$[M-5H]^{5-}$	939.00
$[M-6H]^{6-}$	782.33

Figure S20. LC/MS charts of crude TAMRA-T-modified oligonucleotides with a 3′-aminohexyl and amino-PEG2 linker
